# Visible‐Light‐Driven Fluorescence Turn‐on Photoswitches With Near Quantitative Photocyclization Yield

**DOI:** 10.1002/advs.202414881

**Published:** 2025-01-13

**Authors:** Jiawei Zhai, Qi Ai, Huaqing Li, Zugang Liu, Xiaoguang Hu

**Affiliations:** ^1^ College of Optical and Electronic Technology China Jiliang University No. 258, Xueyuan Street Hangzhou 310018 P. R. China; ^2^ School of Materials Science and Engineering Zhengzhou University No.100 Science Avenue Zhengzhou 450001 P. R. China

**Keywords:** information recording, photoswitch, quantitative photocyclization, turn‐on fluorescence, visible‐light‐driven

## Abstract

Photoswitchable fluorescent materials have gained significant attention for their potential in advanced information encryption and anti‐counterfeiting applications. However, the common use of UV light to trigger the isomerization processes leads to photobleaching and poor fatigue resistance. Visible‐light‐driven fluorescent photoswitches are highly desirable, but achieving high cyclization yield remains challenging. Herein, it is reported that all visible‐light‐driven turn‐on fluorescence in dimethoxyphenyl functionalized diarylethene isomers. The open‐ring form of *o*‐**DMPB** and *p*‐**DMPB** exhibits near quantitative conversion yields (up to 94%) under 405 nm visible light, attributed to the strong electron‐donating character. In contrast, the meta isomer *m*‐**DMPB** shows limited response to visible light, with a ring‐closing yield of 22%. Furthermore, all photoswitches display good thermal stability, photostability, and fatigue resistance. Notably, *o*‐**DMPB** demonstrates promising applications in anti‐counterfeiting, information encryption, and photorewritable patterns. This work provides a valuable strategy for the development of high‐performance fluorescent photoswitches.

## Graphical abstract

It is reported that visible‐light‐driven fluorescence turn‐on photoswitches with near quantitative photocyclization yield. The ortho and para isomers exhibit high ring closing yield of up to 94% upon 405 nm light irradiation, however, that of meta isomer is low to 22%. Moreover, the applications in anti‐counterfeiting, information encryption, and photo rewritable patterns have been demonstrated.

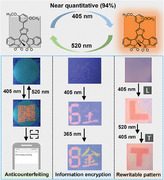

## 1 Introduction

Fluorescent materials have attracted enormous attention in the fields of anti‐counterfeiting and information encryption due to their ease of use, high visibility, diverse emission colors, and difficulty in replication.^[^ ^1^, ^2^, ^3^ ^]^ Traditional fluorescent materials in these applications rely on static fluorescence, where encoded patterns are typically visible under (UV) or near‐infrared (NIR) light.^[^ ^4^, ^5^ ^]^ However, these fluorescent patterns are vulnerable to cloning and counterfeiting using commercially available fluorescent dyes with similar emissive properties, failing to meet the evolving demands of information security.^[^ ^1^, ^6^ ^]^ As a result, it is highly desirable to develop new materials with dynamic fluorescence that can be triggered by external stimuli such as light, heat, chemicals, etc. The added functionality serves as a “key” to activate fluorescence under specific conditions, enhancing information security and making replication more challenging.^[^ ^7^, ^8^, ^9^, ^10^ ^]^ Among these, light‐driven fluorescent photoswitches are promising candidates for constructing dynamic patterns due to their tunable fluorescence intensity, color, reversibility, spatiotemporal resolution, and the simplicity of light as stimuli.^[^ ^3^, ^11^, ^12^, ^13^, ^14^, ^15^ ^]^

To date, numerous fluorescent photoswitches have been developed by combining a photochromic photoswitch with a fluorescent chromophore, the latter will determine the emission color.^[^ ^3^, ^15^, ^16^, ^17^, ^18^ ^]^ Diarylethenes (DAEs) are commonly chosen as the photochromic unit due to their bistability, excellent fatigue resistance, thermal stability, and fast response.^[^ ^16^ ^]^ During the photoisomerization process, the initial fluorescence of the chromophore gradually diminishes, accompanied by a sharp optical color change.^[^ ^3^, ^16^ ^]^ In this way, the unique fluorescent and optical patterns with encoded information can be erased, written, and read reversibly.^[^ ^12^, ^19^, ^20^, ^21^ ^]^ Additionally, Ahn^[^ ^22^, ^23^, ^24^, ^25^, ^26^ ^]^ and Irie^[^ ^27^, ^28^ ^]^ have developed a series of fluorescence turn‐on DAEs by employing electron‐accepting benzothiophene *S*, *S*‐dioxide as central hexatriene units, showing promising potential for applications in information recording and processing materials (e.g., encryption and anti‐counterfeiting).^[^ ^14^, ^29^, ^30^, ^31^ ^]^ However, the photocyclization reactions of most DAEs rely on UV light irradiation,^[^ ^16^ ^]^ which can be harmful as a stimulus, leading to inevitable photodegradation, poor reversibility, and reduced penetrability into samples.^[^ ^16^, ^32^ ^]^

To address this issue, it is of great significance to develop all visible‐light‐driven fluorescence photoswitches. Recent studies on photostability indicate that visible light can improve the photostability of fluorescent DAEs.^[^ ^28^, ^33^, ^34^, ^35^ ^]^ However, the open‐ring form of DAEs rarely undergoes quantitative cyclization to the closed‐ring form under visible light, particularly in fluorescent photoswitches.^[^ ^32^ ^]^ To the best of our knowledge, only a few examples have achieved photocyclization conversion yield exceeding 90%, even under UV light illumination (Table S1, Supporting Information). As a result, fluorescence cannot be fully quenched, leading to low contrast and limiting practical performance. Currently, developing end‐user‐friendly all visible‐light‐driven fluorescent photoswitchable DAEs with high or near quantitative photocyclization conversion remains a significant challenge.^[^ ^28^, ^36^, ^37^, ^38^ ^]^

**Figure 1:**
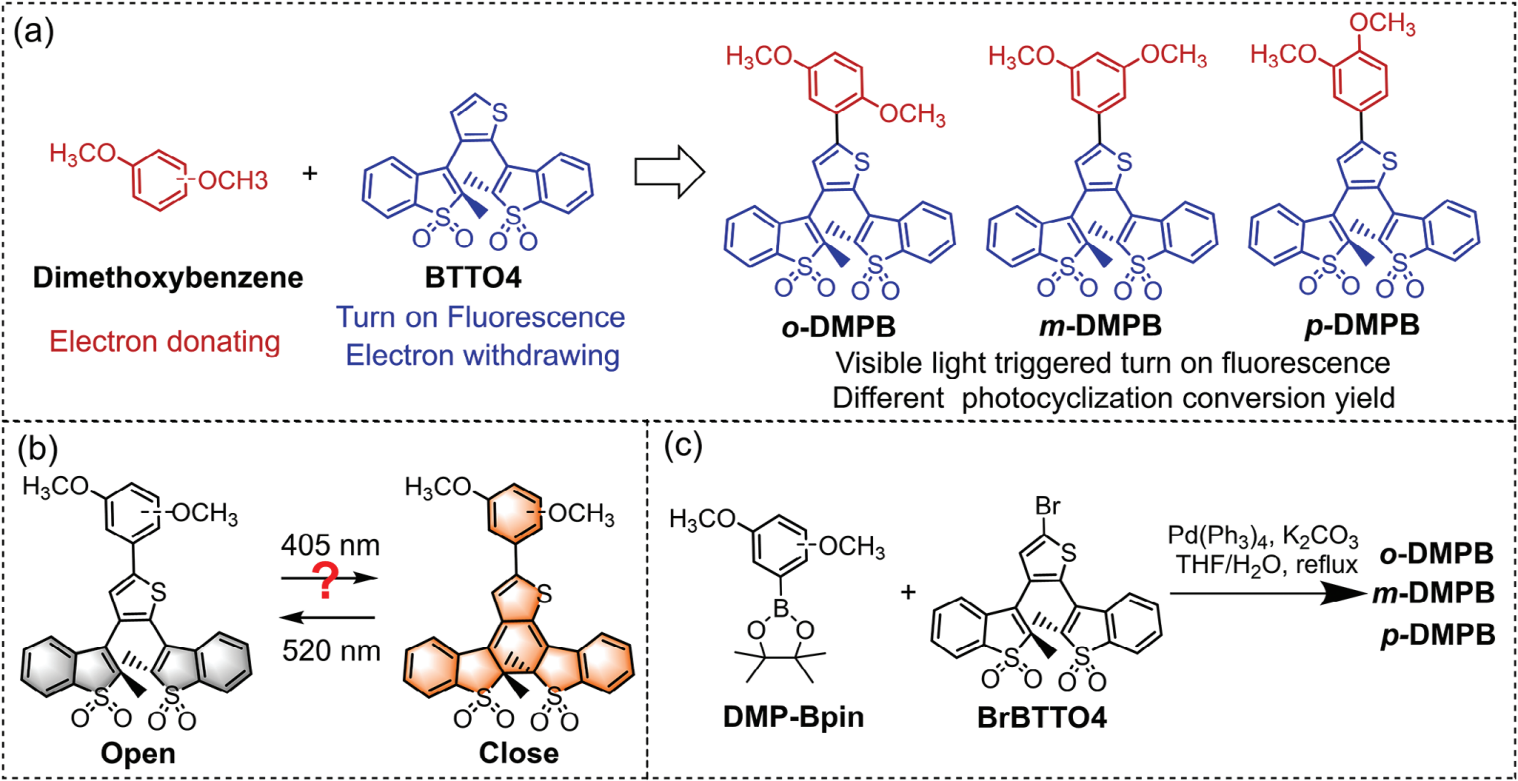
Design strategy, chemical structures a), photochromic reaction upon alternative irradiation of visible light b), and synthetic route c) of *o*‐**DMPB**, *m*‐**DMPB**, and *p*‐**DMPB**.

In order to achieve visible‐light‐driven cyclization of DAEs, the most common strategy is to extend the π‐conjugation, thereby reducing the energy gap between the highest occupied molecular orbital (HOMO) and the lowest unoccupied molecular orbital (LUMO) of open‐ring forms.^[^ ^32^ ^]^ Recently, Li's group^[^ ^39^ ^]^ reported that end‐capping the dithienylethene core with strong electron‐donating groups, such as triphenylamine phenyl groups, enhances its sensitivity to visible light, achieving a 96% photocyclization yield under 405 nm light irradiation. Our group found that introducing electron‐donating groups to BTTO4^[^ ^24^ ^]^ reduces the HOMO‐ LUMO energy gap, enabling visible‐light‐driven fluorescence turn‐on DAEs, and 100% conversion yield was realized in *N*, *N*‐dimethylaniline functionalized derivatives upon 365 nm light irradiation.^[^ ^24^, ^33^, ^34^, ^35^ ^]^ In this study, we designed and synthesized three DAE photoswitches *o*‐**DMPB**, *m*‐**DMPB**, and *p*‐**DMPB** by conjugating BTTO4 with electron‐donating dimethoxyphenyl groups (**Figure** **1**). Upon 405 nm light irradiation, *o*‐**DMPB** and *p*‐**DMPB** underwent near quantitative photocyclization, with conversion yields up to 94%, as confirmed by ^1^H nuclear magnetic resonance, alongside fluorescence turn‐on. In contrast, the meta isomer *m*‐**DMPB** exhibited a low ring‐closing yield (22%) due to its weak electron‐donating capability. Moreover, these photoswitches displayed good thermal stability and photostability, as well as fatigue resistance. Notably, the photoswitchable fluorescence of *o*‐**DMPB** has been applied in anti‐counterfeiting, information encryption, and photo rewritable patterns, showing promising potential for information recording and security.

## 2 Results and Discussion

### 2.1 Molecular Design and Synthesis

The visible‐light‐driven fluorescence turn‐on photoswitchable isomers *o*‐**DMPB**, *m*‐**DMPB**, and *p*‐**DMPB** (Figure 1a) were constructed by conjugating dimethoxyphenyl groups with the UV‐light‐triggered fluorescent turn‐on DAE BTTO4.^[^ ^24^ ^]^ The electron‐donating dimethoxyphenyl extends the conjugation and forms a D‐π‐A conjugation structure with an electron‐withdrawing BTTO4 core, resulting in a red‐shifted absorption edge of open form into the visible light region. The exact absorption depends on the electron‐donating ability of the dimethoxyphenyl groups, with the ortho and para isomers generally being stronger than meta isomers. Thus, the sensitivity to visible light and the photocyclization conversion yield under the same wavelength are dependent on substituents (Figure 1b). Additionally, the photocyclization yield can be enhanced by conjugating phenyl groups to BTTO4 core.^[^ ^24^, ^33^, ^34^, ^35^ ^]^ The starting materials, dimethoxyphenyl boronic acid pinacol esters (DMP‐Bpin), are commercially available, while BrBTTTO4 was prepared according to the literature.^[^ ^24^ ^]^ The target molecules were then obtained via a Suzuki cross‐coupling reaction (Figure 1c), with the detailed procedure described in the Supporting Information. The chemical structures of the target molecules were confirmed by ^1^HNMR, ^13^CNMR, and high‐resolution mass spectrometry (Figures S1–S9, Supporting Information).

### 2.2 Photochromic Properties

**Figure 2:**
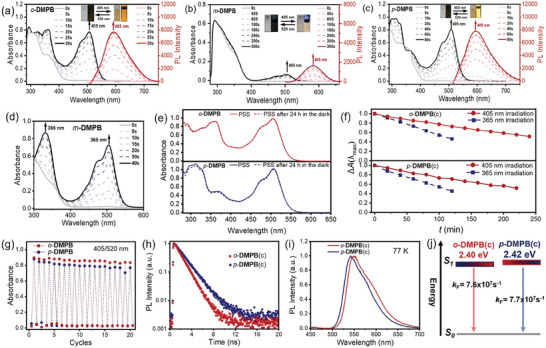
Changes in the absorption and fluorescence spectra of *o*‐**DMPB** a), *m*‐**DMPB** b), and *p*‐**DMPB** c) in toluene (5.0 × 10^−5^ m) upon irradiation with visible light at 405 nm. Excited at 405 nm, slit widths: 2.5 nm and 2.5 nm. Insets: corresponding color changes between open form and photo stationary state under UV and daylight. d) Absorption spectra changes of *m*‐**DMPB** in toluene (5.0 × 10^−5^ m) upon irradiation with UV (365 nm). e) Absorption spectra of *o*‐**DMPB**(c) and *p*‐**DMPB**(c) in toluene (≈5.0 × 10^−5^ m) before and after stored in the dark for 24 h. f) Photostability of *o*‐**DMPB**(c) and *p*‐**DMPB**(c) in toluene (5.0 × 10^−5^ m) under continued 365 and 405 nm light illumination. g) Absorbance switching of *o*‐**DMPB** and *p*‐**DMPB** in toluene (≈5.0 × 10^−5^ m) monitored at λ_max_ upon alternative irradiation with 405 nm and 520 nm. h) Transient decay spectra of *o*‐**DMPB**(c) and *p*‐**DMPB**(c) in toluene at room temperature. i) Fluorescence spectra of *o*‐**DMPB**(c) and *p*‐**DMPB**(c) in toluene at 77 K. j) Energy level diagram of *o*‐**DMPB**(c) and *p*‐**DMPB**(c). The intensity of 365, 405, and 520 nm light for the photochromic reaction is ≈1.69, 1.17, and 2.82 mW cm^−2^ via optical power meter detection, and the distance between the light source and solution sample (cuvette) was 10 cm.

Photochromic properties of *o*‐**DMPB**, *m*‐**DMPB**, and *p*‐**DMPB** were studied in toluene. As depicted in **Figure** **2**a,c, *o*‐**DMPB**(o) and *p*‐**DMPB**(o) show broad absorption bands (λ_abs_) centered at 368 and 356 nm, with molar excitation coefficients (ε) of 7.5 and 9.4 mM^−1^ cm^−1^, respectively. As expected, the introduction of dimethoxyphenyl groups red‐shifts the absorption edge of BTTO4(o) (≈ 360 nm)^[^ ^24^ ^]^ to the visible light region (> 410 nm). Whereas, the meta isomer *m*‐**DMPB**(o) displays a shorter absorption edge ≈408 nm due to the weak electron donating ability of the meta methoxy group. Upon 405 nm visible light irradiation, a broad absorption band appeared in the visible light range between 400 and 550 nm, revealing the formation of closed‐ring forms and the activation of turn‐on fluorescence. The photostationary state (PSS) was reached after 30 and 80 s of irradiation for *o*‐**DMPB**(o) and *p*‐**DMPB**(o), respectively, with strong absorption and emission peaks at 506/593 nm and 508/596 nm. In contrast, *m*‐**DMPB** is less responsive to 405 nm light, even after 360 s irradiation, the PSS was reached with weak absorption in the visible light region, showing λ_max_ at 504 nm and emission intensity at 582 nm, as evidenced by the faint color and fluorescence images in the inset of Figure 2b. These changes in color and fluorescence in the closed forms are consistent with the corresponding spectra. When irradiated with visible light longer than 450 nm (a 520 nm visible light lamp was used in this study), these closed‐ring forms underwent cycloreversion, returning to their open‐ring forms and restoring the original absorption and emission spectra. The photochromic properties of *m*‐**DMPB** were also investigated under UV light. As shown in Figure 2d, this compound is highly responsive to UV light, upon 365 nm light irradiation, strong absorption in the visible light region appeared, and the PSS was reached after 40 s of continued irradiation.

The stability and fatigue resistance of these compounds were then evaluated, which are beneficial for practical application. The absorption intensities at λ_max_ in the PSS remained nearly unchanged after being stored in the dark at ambient temperature for 24 h, indicating excellent thermal stability (Figure 2e; S19, Supporting Information). This result suggests that with the introduction of the dimethoxy group, the molecules still maintain a relatively larger activation energy difference between open and closed‐ring forms in the ground state.^[^ ^40^, ^41^ ^]^ To demonstrate the advantage of visible‐light‐triggered photocyclization, photostability was studied under prolonged irradiation with 365 and 405 nm light, respectively (Figure 2f). Obviously, the degradation of the closed‐ring form of *o*‐**DMPB** and *p*‐**DMPB** under 405 nm light irradiation was significantly slower than that under 365 nm light. Thus, visible light could reduce side reactions and enhance photostability during the photocyclization process. Additionally, all photoswitches exhibited good fatigue resistance, showing no apparent loss of photochromic activity after 20 photocyclization and photocycloreversion cycles under alternate 405 (Figure 2g) /365 nm (Figure S20, Supporting Information) and 520 nm light irradiation.

### 2.3 Turn‐on Fluorescence Properties

Fluorescence emission at 77 K, photoluminescence quantum yields (PLQYs), and transient fluorescence decay were measured for the closed‐ring forms to investigate the fluorescence turn‐on properties. Since *m*‐**DMPB**(o) is insensitive to visible light, its fluorescent properties were not further studied. At room temperature (RT) under an N_2_ atmosphere, the PLQYs of *o*‐**DMPB**(c) and *p*‐**DMPB**(c) were measured as 0.13 and 0.10, respectively. Their transient decay spectra displayed a single exponential feature with fluorescence lifetimes of 1.29 and 1.72 ns (Figure 2h), respectively. When cooled to 77 K, the emission of *o*‐**DMPB**(c) and *p*‐**DMPB**(c) blue shifted to shorter wavelengths (≈550 and 542 nm) (Figure 2i), indicating that the non‐radiative decay at RT in toluene is closely related to the excited‐state geometric relaxation process. From the onset of the fluorescence spectra in 77 K, the S_1_ energies of *o*‐**DMPB**(c) and *p*‐**DMPB**(c) were estimated to be 2.40, and 2.42 eV, respectively (Figure 2j). In fluid solutions, it is widely known that the solvation and relaxation processes of excited states are completed within a few picoseconds,^[^ ^42^, ^43^ ^]^ leading to rapid non‐radiative decay through free rotor and loose bolt effects.^[^ ^44^ ^]^ The fluorescence radiative rate (*k*_F_) value can be obtained by the formula: *k*_F_ = Φ_F_/τ, where Φ_F_ and τ represent the PLQY and fluorescence lifetime, respectively. The *k*_F_ values for these two molecules were calculated to be ≈7.7 × 10^7^ s^−1^. Recent studies have suggested that TADF emitters with high *k*_F_ may achieve efficiency and stability by suppressing the singlet‐singlet annihilation (SSA) process.^[^ ^42^ ^]^ In this study, over 80% of absorbance intensity was retained after 405 nm irradiation for 85 min, suggesting that the relatively high *k*_F_ contributes to improved photostability.

### 2.4 Photocyclization Conversion Yield

**Figure 3:**
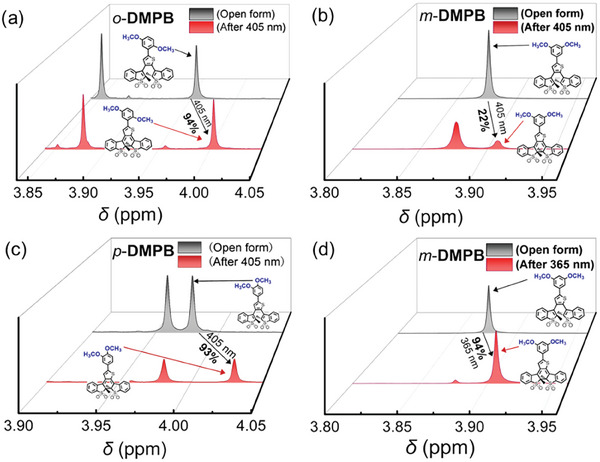
The changes in the chemical shift of the methoxy groups in ^1^HNMR spectra (CDCl_3_) of *o*‐**DMPB** a), *m*‐**DMPB** b,d), and *p*‐**DMPB** c) before (gray area) and after (red area) 405 or 365 nm light irradiation indicate a photocyclization reaction yield of 94%, 22%, 93%, and 94%, respectively.

In the previous report, 100% conversion yield was achieved in *N*, *N*‐dimethylaniline functionalized BTTO4 derivatives upon 365 nm light irradiation. However, the conversion yield under visible light was not measured.^[^ ^33^ ^]^ To determine the photocyclization reaction yield of these photoswitches under 405 nm irradiation, the ^1^H NMR spectra were recorded (**Figure** **3**). In CDCl_3_, the chemical shift of the methyl groups on the active carbon makes it difficult to distinguish between open‐ and closed‐ring forms, therefore, the dimethoxy groups were chosen for calculating the cyclization ratio. For *o*‐**DMPB**(o), the chemical shift of the methoxy groups was 3.85 and 3.96 ppm. Upon 405 nm light irradiation, new peaks corresponding to the closed‐ring form appeared at 3.87 and 4.00 ppm, allowing the cyclization yield to be calculated as 94% (Figure 3a). To the best of our knowledge, this is the first all visible‐light‐driven fluorescence turn‐on DAE with a near quantitative photocyclization conversion yield under 405 nm light irradiation, which has rarely been reported in the literature (Table S1, Supporting Information). When irradiated with 365 nm UV light, near quantitative conversion (95%) was also achieved for *o*‐**DMPB** (Figure S12, Supporting Information). For *m*‐**DMPB** and *p*‐**DMPB** (Figures 3b,c), photocyclization conversion yields of 22% and 93%, respectively, were estimated under 405 nm light irradiation based on changes in ^1^HNMR spectra. Upon switching the light source to UV light (365 nm), the response is enhanced and the photocyclization yield of *m*‐**DMPB** gradually increases to 94% (Figure 3d). Thus, conjugating electron‐donating groups with BTTO4 proves to be an effective strategy for achieving near quantitative photocyclization yield under either visible (e.g., 405 nm) or UV (e.g., 365 nm) light. Consequently, 405 nm visible light could serve as an alternative to UV light for driving the photocyclization reaction in fluorescent photoswitches.

### 2.5 Theoretical Calculation

**Figure 4:**
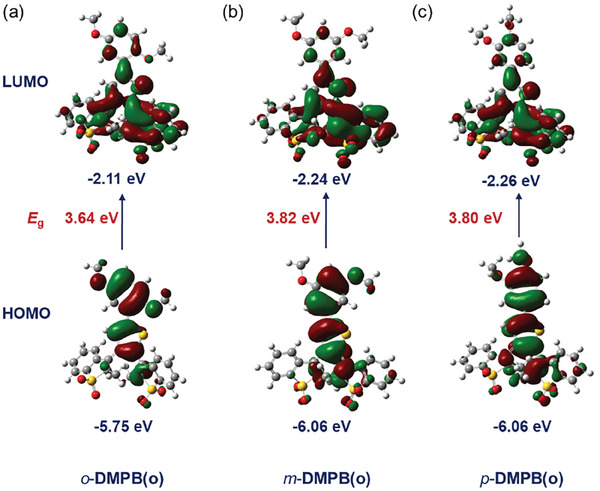
The highest occupied and lowest unoccupied molecular orbitals (HOMO and LUMO) of the open‐ring forms of *o*‐**DMPB** a), *m*‐**DMPB** b), and *p*‐**DMPB** c) at the ground state.

To gain deeper insight into the photophysical properties of these DAEs, density functional theory (DFT) and time‐dependent DFT (TD‐DFT) calculations were performed using the B3LYP/6‐31G(d) basis. The calculated distance between the two active carbon atoms of these open‐isomers (≈ 3.6 Å, Figure S28, Supporting Information) is short enough to facilitate photocyclization.^[^ ^16^ ^]^ Compared to the open‐ring form of the reported photoswitch Ph‐BTTO4 (*E*_g_ = 3.91 eV, absorption edge ≈395 nm) without the dimethoxy group,^[^ ^24^ ^]^ the HOMO‐LUMO energy gaps of *o*‐**DMPB** and *p*‐**DMPB** were reduced to 3.64 and 3.80 eV, respectively, due to the strong electron‐donating character of the ortho and para dimethoxy group (**Figures** **4**a,c). This reduction in the energy gap suggests a red shift in the absorption edge, allowing visible‐light‐driven cyclization. In contrast, the 3,5‐dimethoxyphenyl group in *m*‐**DMPB**(o) (*E*_g_ = 3.82 eV, Figure 4b), exhibits a slightly larger energy gap compared to *o*‐**DMPB**(o) and *p*‐**DMPB**, as its p‐π conjugation is less effective. These calculated energy gaps correlate with the photoswitches' sensitivity to 405 nm light and their photocyclization conversion yields. Additionally, the energy of vertical absorption (S_VA_) and oscillator strengths (*f*) were also calculated by time‐dependent DFT (TD‐DFT) with optimized closed‐isomer structures (Table S3, Supporting Information). The estimated S_VA_ values for *o*‐**DMPB**(c) (2.42 eV), *m*‐**DMPB**(c) (2.47 eV), and *p*‐**DMPB**(c) (2.47 eV) are slightly smaller than that of Ph‐BTTO4(c) (2.48 eV), with similar *f* values (≈ 0.35), indicating their turn‐on fluorescence properties and longer emission maxima in comparison with Ph‐BTTO4(c). Thus, the molecular design strategy successfully enables visible‐light‐driven photoswitches with near‐quantitative photocyclization yields and turn‐on fluorescence.

### 2.6 Applications

**Figure 5:**
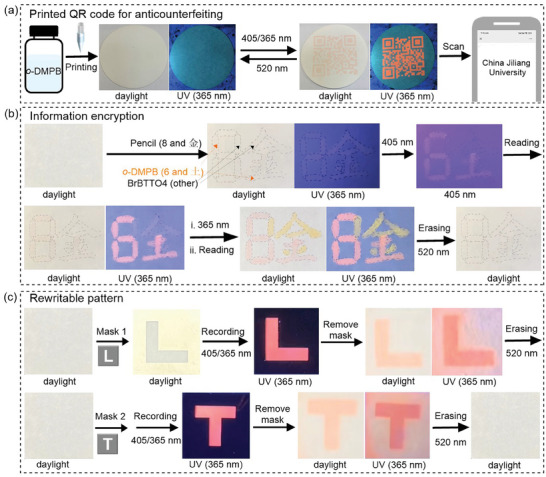
All visible‐light‐driven information recording applications of *o*‐**DMPB**. a) Reversible anti‐counterfeiting behavior: Photographs of encoded information printed on filter paper (D9 cm) upon alternating 405 (or 365) and 520 nm light irradiation under daylight and 365 nm UV lamp. b) Information encryption and anti‐counterfeiting: *o*‐**DMPB** (visible light sensitive) and BrBTTO4 (visible light insensitive) were coated onto separate sections of a number “8” and Chinese character “金” pattern which appears in sequence when sequentially exposing the whole sample to 405 nm light and then 365 nm light. c) Rewritable pattern recording: Writing, reading, and erasing processes of two letters on *o*‐**DMPB** coated filter paper using alternating 405 and 520 nm light irradiations with the aid of masks.

The above results demonstrated that fluorescence turn‐on *o*‐**DMPB** and *p*‐**DMPB** exhibit excellent thermal stability and photostability, fatigue resistance, and near quantitative photocyclization yield under visible light irradiation, enabling them promising for practical applications. *o*‐**DMPB**, in particular, was employed as a fluorescent photoswitch for information recording and security purposes. A prototype was provided to showcase its potential application in anti‐counterfeiting. 10 mg mL^−1^ colorless ink of *o*‐**DMPB** in the mixed solvent of 80% butyl benzoate and 20% toluene was formulated, and then the encoded information was printed on filter papers by the HS Electronics IJDAS‐300 Printer with paint nozzle DMC‐11610 and a dpi (dots per inch) of 400. As shown in **Figure** **5**a, the printed security code is invisible under both daylight and UV light due to the colorless and non‐fluorescence nature of open‐isomer. After ≈60 s irradiation with a 405 nm lamp, the printed quick response (QR) code became optically visible and fluorescently emissive, as *o*‐**DMPB**(o) switched to the closed form. Impressively, the QR code could be easily recognized and read under UV light using a camera, while the words “China Jiliang University” were discernible after scanning with commercially available apps like “WeChat” or “Alipay”, both of which have over one billion users. Furthermore, the pattern can be erased and returned to its original invisible state by irradiating it with a 520 nm lamp for 90 s, and the recording and erasing processes can be repeated for 20 cycles without significant degradation. These proof‐of‐concept demonstrations highlight the potential of *o*‐**DMPB**‐based ink for anti‐counterfeiting applications through inkjet printing technology.

Moreover, benefiting from the visible‐light‐driven photocyclization, *o*‐**DMPB** could function for information encryption (Figure 5 b). Initially, the number “8” and the Chinese character “金” were written on a filter pattern using a pencil. *o*‐**DMPB** was then applied to cover the region containing the number “6” and the Chinese character “土”, while the remaining area was coated with the starting material BrBTTO4, for which the cyclization was only triggered under UV light. Similarly, both regions remained invisible under daylight and UV light. However, upon being illuminated with 405 nm light, the number “6” and the Chinese character “土” appeared under daylight and UV light due to the photocyclization of *o*‐**DMPB**. When irradiated with 365 nm light, the entire pattern, including “8” and “金”, became visible and emitted fluorescence, as BrBTTO4 cyclized to the closed form. Notably, the yellow emission of BrBTTO4(c) differed from that of *o*‐**DMPB**. As expected, the pattern could be erased by irradiating it with 520 nm visible light. Thereby, an information encryption prototype was demonstrated based on *o*‐**DMPB** and BrBTTO4 (Video S1, Supporting Information).

In addition, *o*‐**DMPB** can be used as photo‐rewritable information recording materials due to its photochromism on filter paper (Figure 5c). First, the filter paper was dipped into an *o*‐**DMPB** solution in ethyl acetate, then taken out and air‐dried. Next, a mask containing the letter “L” was placed onto the treated filter paper and illumination with 405 or 365 nm light, transferring the mask's letter onto the paper. The optically visible and fluorescent “L” appeared once the mask was removed. The recorded letter was then completely erased after exposure to 520 nm visible light. The photo writing and erasing process could be repeated utilizing another mask, e.g., the letter “T” or a QR code with encrypted information. Thus, the prepared DAEs can function as photoswitch to construct photo‐rewritable fluorescence patterns that can be frequently changed or encrypted.

## 3 Conclusion

In summary, we have successfully designed and synthesized three photoswitches, *o*‐**DMPB**, *m*‐**DMPB**, and *p*‐**DMPB**, exhibiting visible‐light‐driven turn‐on fluorescence. The *o*‐**DMPB** and *p*‐**DMPB** derivatives, featuring a D‐π‐A conjugation structure and strong electron‐donating ortho and para dimethoxyphenyl groups, achieved nearly quantitative photocyclization with conversion yields reaching up to 94% upon 405 nm light irradiation. This represents the highest reported value among fluorescence turn‐on DAEs. In contrast, the meta isomer *m*‐**DMPB** demonstrates a significantly lower ring closing yield of 22% under identical conditions, attributed to its weaker electron‐donating properties. Additionally, all these photoswitches display good thermal stability, photostability, and fatigue resistance. Leveraging these properties, *o*‐**DMPB** has been effectively applied in anti‐counterfeiting, information encryption, and photo‐rewritable patterns applications, highlighting its potential for advanced optical technologies. This study presents a robust strategy for achieving near‐quantitative photocyclization yields under visible light irradiation in fluorescent DAEs. Ongoing research in our laboratory aims to extend the absorption range of open‐ring form and enhance the fluorescence quantum yield of closed‐ring form while maintaining their superior photochromic properties.

## Conflict of Interest

The authors declare no conflict of interest.

## Supporting information



Supporting Information

Supplemental Video 1

## Data Availability

The data that support the findings of this study are openly available in [AQ] at [https://doi.org], reference number [10000].
