# Supplementary material for: Visible‐Light‐Driven Fluorescence Turn‐on Photoswitches With Near Quantitative Photocyclization Yield
Source: Adv Sci (Weinh). 2025 Jan 13;12(9):2414881. doi: 10.1002/advs.202414881 (PMC11884615; doi:10.1002/advs.202414881)
Supplement: Supplementary file 1 — Supporting Information [file ADVS-12-2414881-s002.docx]

Supporting Information:

Visible-Light-Driven Fluorescence Turn-on Photoswitches with Near Quantitative Photocyclization Yield

Jiawei Zhai^a^, Qi Ai^a*^, Huaqing Li^b^, Zugang Liu^a*^ and Xiaoguang Hu^b*^

^a^ College of Optical and Electronic Technology, China Jiliang University, Hangzhou 310018, P. R. China. E-mail: aiqi@ cjlu.edu.cn; zgliu78@cjlu.edu.cn

^b^ School of Materials Science and Engineering, Zhengzhou University, Zhengzhou 450001, P. R. China. E-mail: xghu@zzu.edu.cn

Table of Contents

[1. General Methods and Materials 3](#_Toc177977920)

[2. Summary of photocyclization conversion yield of reported fluorescent DAEs 4](#_Toc177977921)

[3. Synthetic details 8](#_Toc177977922)

[4. Photophysical properties 14](#_Toc177977923)

[5. Theoretical calculation 29](#_Toc177977924)

[6. References 30](#_Toc177977925)

[7. Appendix 31](#_Toc177977926)

1. General Methods and Materials

All reagents were purchased from Macklin, Aladdin, TCI, and Adamas and used as received. Column chromatography was performed with General-Reagent silica gel (300-400 mesh). All reaction mixtures and column eluents were monitored by TLC using commercial Huanghai glass plates (HSGF 254, 2.5×8 cm). The 405 nm irradiation experiment was carried out using a 5W ANJOET 405 nm flashlight with a filter from DHC (405 nm, T≥85%, FWHM10nm GCC-201008). 310 nm irradiation experiment was carried out using a Shanghai Baoshan Gucun Photoelectricity Instrument ZF-7D (16 W, 50 Hz) 310 nm lamp The intensity of 310 nm light for photochromic reaction is approximately 0.80 mW/cm^2^ via optical power meter detection, the distance between light source and solution sample (cuvette) was 10 cm. 365 nm and 520 nm irradiation experiment were carried out using Anfu 365 and 520 nm flashlight (~ 8W). The intensity of the light source was measured using a Beijing China Education Au-light Co., Ltd CEL-NP2000-2 optical power meter. The absorption spectra were recorded using a Shimadzu UV-2600 spectrophotometer. The room temperature ﬂuorescence spectra were performed by a Techcomp FL-970 Plus spectrometer. The low-temperature fluorescence spectra were collected by the strobing technique and an electric shutter, respectively, using the PTI QM-40 spectrofluorometer equipped with a PTI nitrogen laser (GL-3300, λ=266 nm, pulse width∼1 ns, pulse energy=1.45 mJ) and a liquid nitrogen dewar. The photoluminescence quantum yields were obtained by the QM-40 equipped with an integrating sphere attachment. The transient decay spectra were measured by the TCSPC technique, using a Horiba DeltaFlex modular lifetime measurement system equipped with a diode laser (λ=371 nm, pulse width≈50 ps, repetition rate=20.00 kHz). The fluorescence decay curves were analyzed using the deconvolution software. NMR spectra were measured by a Bruker AV III HD 400 MHz. High resolution mass spectra (HRMS) were measured on an ACQUITY UPLC I-CLASS (ESI) and DANI MASTER GCXGC TOF-MS.

2. Summary of photocyclization conversion yield of reported fluorescent DAEs

**Table S1**. The photocyclization conversion yield of reported fluorescent DAEs.

| Molecular structures | *α*PSS@λ_irr_ (nm)  /Measurement methods | Fluorescence mode (quantum yield) | Visible-light response  mechanism | References |
| --- | --- | --- | --- | --- |
|  | 82%@365 nm  HPLC | Turn-on |  | Adv. Funct. Mater*.,* 2018, 28, 1706213 |
|  | 98%@313 nm  absorption spectrum | Turn-on  0.12% |  | Chinese Chemical Letters, 32 (2021), 3882-3885 |
|  | 89.5%@365 nm  HPLC, absorption spectrum | Turn-on  29.9% |  | J. Mater. Chem. C, 2022, 10, 8860-8873 |
|  | 100%@365 nm  HPLC | Turn-on  40% |  | J. Am. Chem. Soc., 2022, 144, 14235-14247 |
|  | 100%@365 nm  HPLC | Turn-on  41% |  | J. Am. Chem. Soc., 2022, 144, 14235-14247 |
|  | 405 nm  No information | Turn-on  74-78% | Extend π-Conjugation | Chem. Commun., 2022, 58, 4715-4718 |
|  | 405 nm  No information | Turn-on  42% | Extend π-Conjugation | Photochem. Photobiol. Sci., 2020, 19, 783-789 |
|  | 89%@365 nm  HNMR | Open form 50%  Closed form 40% |  | Chem. Commun., 2020, 56, 2198-2201 |
|  | 83%@312 nm  HNMR | Turn-on  3.6% |  | Bull. Korean Chem. Soc., 2018, DOI: 10.1002/bkcs.11597 |
|  | 97%@312 nm  HNMR | Turn-on  0.6% |  | Bull. Korean Chem. Soc., 2018, DOI: 10.1002/bkcs.11597 |
|  | 82%@312 nm  HNMR | Turn-on  2.4% |  | Chem. Eur. J., 2016, 22, 656-662 |
|  | 97%@365 nm (**6-Et-H**)  90%@365 nm (**6-iBu-H**)  99%@365 nm (**7-Et-H**)  93%@365 nm (**7-iBu-H**)  96%@365 nm (**6-Et-OMe**)  78%@365 nm (**6-iBu-OMe**)  95%@365 nm (**7-Et-OMe**)  75%@365 nm (**7-iBu-OMe**)  HPLC, absorption spectrum | Turn-on  44% (**6-Et-H**)  46%(**6-iBu-H**)  46% (**7-Et-H**)  46%(**7-iBu-H**)  30%(**6-Et-OMe**)  34%(**6-iBu-OMe**)  33%(**7-Et-OMe**)  34%(**7-iBu-OMe**) |  | J. Am. Chem. Soc., 2019, 141, 16471-16478 |
|  | ~100%@313 nm  >65%@405 nm  HPLC | 80% |  | Org. Lett., 2015, 17, 4802-4805 |
|  | ~100%@365 nm  HNMR  405 nm (no information) | Turn-on  8.87% for mDB  2.73% for pDB | Extending π conjugation, donor-accepter structure | Dyes and Pigments, 204 (2022), 110441 |
|  | ~100%@365 nm | Turn-on  75% |  | Chem. Sci., 2016, 7, 5867-5871 |
|  | 92%@313 nm | Turn-on  5% |  | Dyes and Pigments, 126 (2016), 186e193 |
|  | >99%@313 nm | Turn-on  35% |  | J. Mater. Chem., 2011, 21, 17425-17432 |
|  | ~80%@313 nm | Turn-on  37% |  | J. Mater. Chem., 2011, 21, 17425-17432 |
|  | >99%@313 nm | Turn-on  3% |  | J. Mater. Chem., 2011, 21, 17425-17432 |
|  | ~80%@313 nm | Turn-on  3% |  | J. Mater. Chem., 2011, 21, 17425-17432 |
|  | ~65%@313 nm  HPLC | Turn-on  11% |  | Chem. Commun., 2012, 48, 3745-3747 |
|  | 81.0%@532 nm  /absorption spectrum | Turn-on | TTA-UC | Chem. Eur. J., 2023, 29,  e202203651 |
|  | 74.8%@365 nm  HNMR  405 nm (no information) | Turn-on  21% | Extending π conjugation, donor-accepter structure | Dyes and Pigments, 205 (2022), 110571 |
|   | 405 nm (no information) | Turn-on  11-19% | Extending π conjugation, donor-accepter structure | Journal of Luminescence, 248 (2022), 118973 |
|  | 53.2%@365 nm  HNMR  405 nm (no information) | Turn-on  5% | Extending π conjugation, donor-accepter structure | Acta. Chim. Sinica., 2022, 80, 1223-1230 |
| 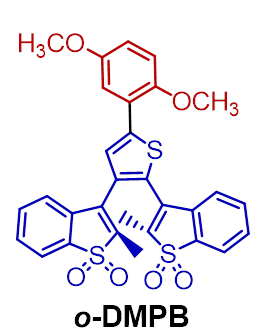 | 94%@405 nm  HNMR | Turn-on  10% | Extending π conjugation, donor-accepter structure | This work |
| 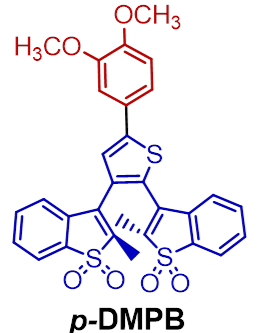 | 93%@405 nm  HNMR | Turn-on  13% | Extending π conjugation, donor-accepter structure | This work |
|  | ~67%@297 nm  HPLC | Turn off  36% |  | Spectrochimica Acta Part A: Molecular and Biomolecular Spectroscopy, 138 (2015), 441-446 |
|  | 90.0%@532 | Turn off | TTET | Chem. Lett., 2022, 51, 1095-1098 |
|  | 50%@405  86%@365  /HNMR | Turn off | Extend π-Conjugation | ACS Appl. Mater.  Interfaces, 2020, 12, 27651-27662 |
|  | 90.5%@460  /HPLC | Turn off | Extend π-Conjugation | Dyes and Pigments, 2020, 179, 108419 |
|  | 91.4%@460  /HPLC | Turn off | Extend π-Conjugation | Dyes and Pigments, 2020, 182, 108686 |
|  | 97%@560 (o→c) | Turn off | Extend π-Conjugation | J. Am. Chem. Soc., 2014, 136, 17145-17154 |
|  | 79%@402  73%@302  /HPLC | Turn off | Extend π-Conjugation | J. Org. Chem., 2015, 80, 7830-7835 |

3. Synthetic details

Synthesis of *o***-DMPB**

To a stirred solution of BrBTTO4 (150.64 mg, 0.29 mmol) in tetrahydrofuran (10 mL), (2,5-dimethoxyphenyl)boronic acid (63.69 mg, 0.35 mmol), saturated aqueous K_2_CO_3_ solution (5 mL), and Pd(PPh_3_)_4_ (16.75 mg, 0.05 equivalent) were added. The mixture was degassed with argon for approximately 15 minutes and then refluxed for 8 hours. The reaction mixture was cooled and extracted with ethyl acetate. The combined organic layer was washed with water, followed by brine solution and drying over anhydrous MgSO_4_. This organic layer was filtered off and concentrated to give the crude product, which was purified by column chromatography (stationary phase: silica gel 60-120, mobile phase: DCM/PE=1:3) to give *o***-DMPB** (142.00 mg, 85%) as an orange solid. ^1^H NMR (400 MHz, CDCl_3_): *δ* [ppm] 1.88-2.22 (m, 6H), 3.95-4.01 (m, 6H), 6.92-6.95 (m, 1H), 7.01 (d, *J*=8 Hz, 1H), 7.19-7.25 (m, 2H), 7.47-7.60 (m, 5H), 7.65 (s, 1H), 7.75 (d, *J*=4 Hz, 2H); ^13^C NMR (100 MHz, CDCl_3_): *δ* [ppm] 153.88, 150.07, 143.10, 133.47, 132.36, 130.81, 129.82, 129.71, 126.50, 122.87, 121.83, 121.81, 121.78, 114.81, 113.57, 113.05, 56.27, 55.95, 8.49, 8.17. HRMS [M+H]^+^ calcd. for C_30_H_25_O_6_S_3_: 577.0813, found 577.0800.

Synthesis of *m***-DMPB**

A procedure similar to that used for *o*-**DMPB** produced *m*-**DMPB** as an orange solid in 77% yield. ^1^H NMR (400 MHz, CDCl_3_): *δ*[ppm] 1.83-2.12 (m, 6H), 3.79 (s, 6H), 6.45 (t, *J*=10 Hz, 1H), 6.72 (d, *J*=2 Hz, 2H), 7.18-7.2 (m, 1H), 7.38-7.45 (m, 6H), 7.63-7.66 (m, 2H); ^13^C NMR (100 MHz, CDCl_3_): *δ* [ppm] 161.49, 148.221, 134.10, 132.17, 132.13, 129.95, 129.81, 124.80, 122.87, 121.89. 121.84, 104.61, 100.80, 55.61, 8.49, 8.18. HRMS [M+H]^+^ calcd. for C_30_H_25_O_6_S_3_: 577.0813, found 577.079.

Synthesis of *p***-DMPB**

A procedure similar to that used for *o***-DMPB** produced *p***-DMPB** as an orange solid in 80% yield. ^1^H NMR (400 MHz, CDCl_3_): *δ* [ppm] 1.88-2.21 (m, 6H), 3.97 (d, *J*=8 Hz, 6H), 6.96 (d, *J*=8 Hz, 1H), 7.12-7.64 (m, 9H), 7.75-7.82 (m, 2H); ^13^C NMR (100 MHz, CDCl_3_): *δ* [ppm] 150.12, 149.54, 148.45, 132.19, 129.92, 129.79, 125.31, 123.57, 121.91, 119.04, 111.65, 109.21, 56.15, 56.11, 8.54, 8.22. HRMS [M+H]^+^ calcd. for C_30_H_25_O_6_S_3_: 577.0813, found 577.0806.

**Figure S1.** ^1^H NMR spectra of *o***-DMPB**.

**Figure S2.** ^13^C NMR spectra of *o***-DMPB**.


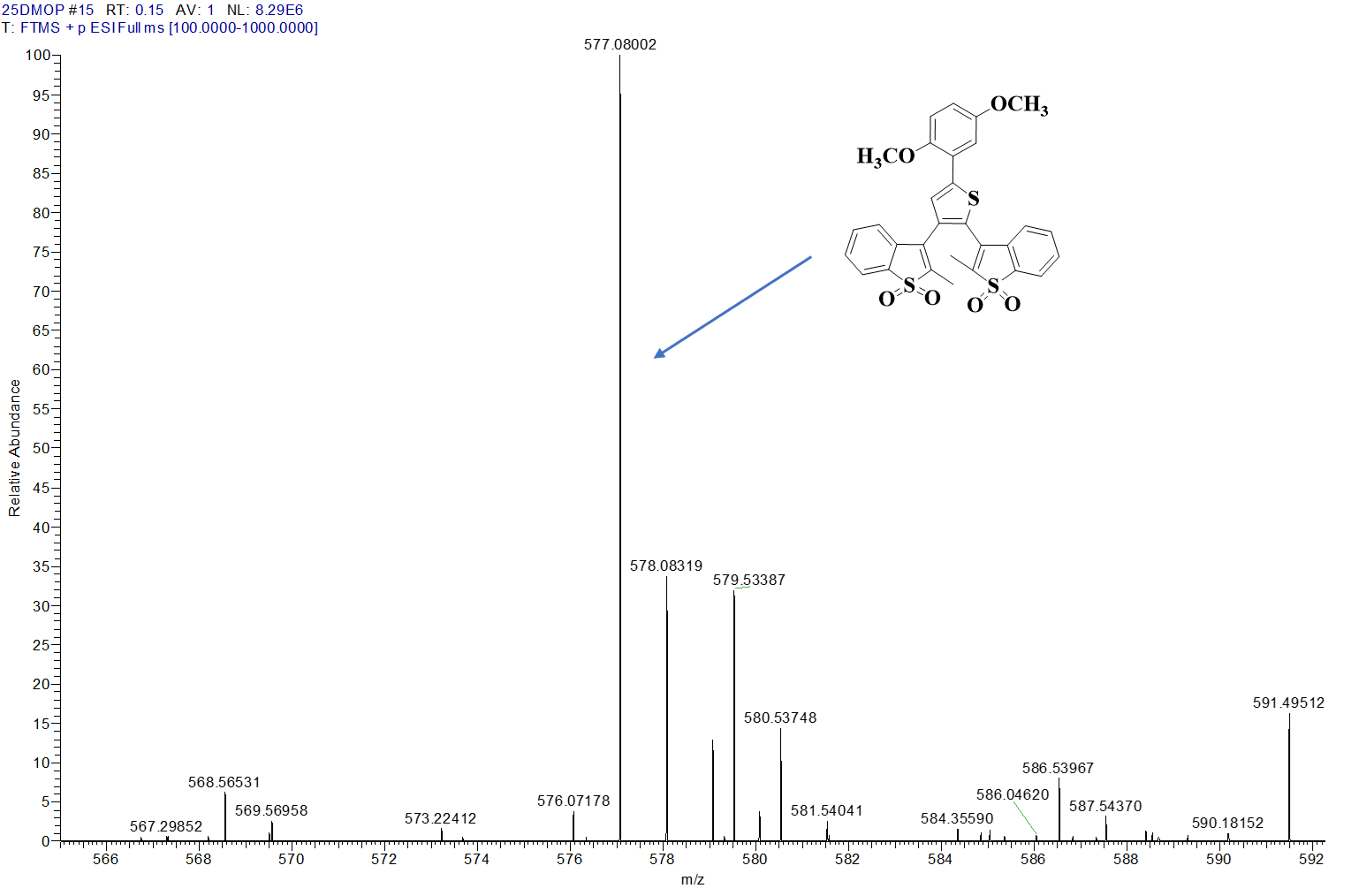


**Figure S3.** HRMS spectra of *o***-DMPB**.


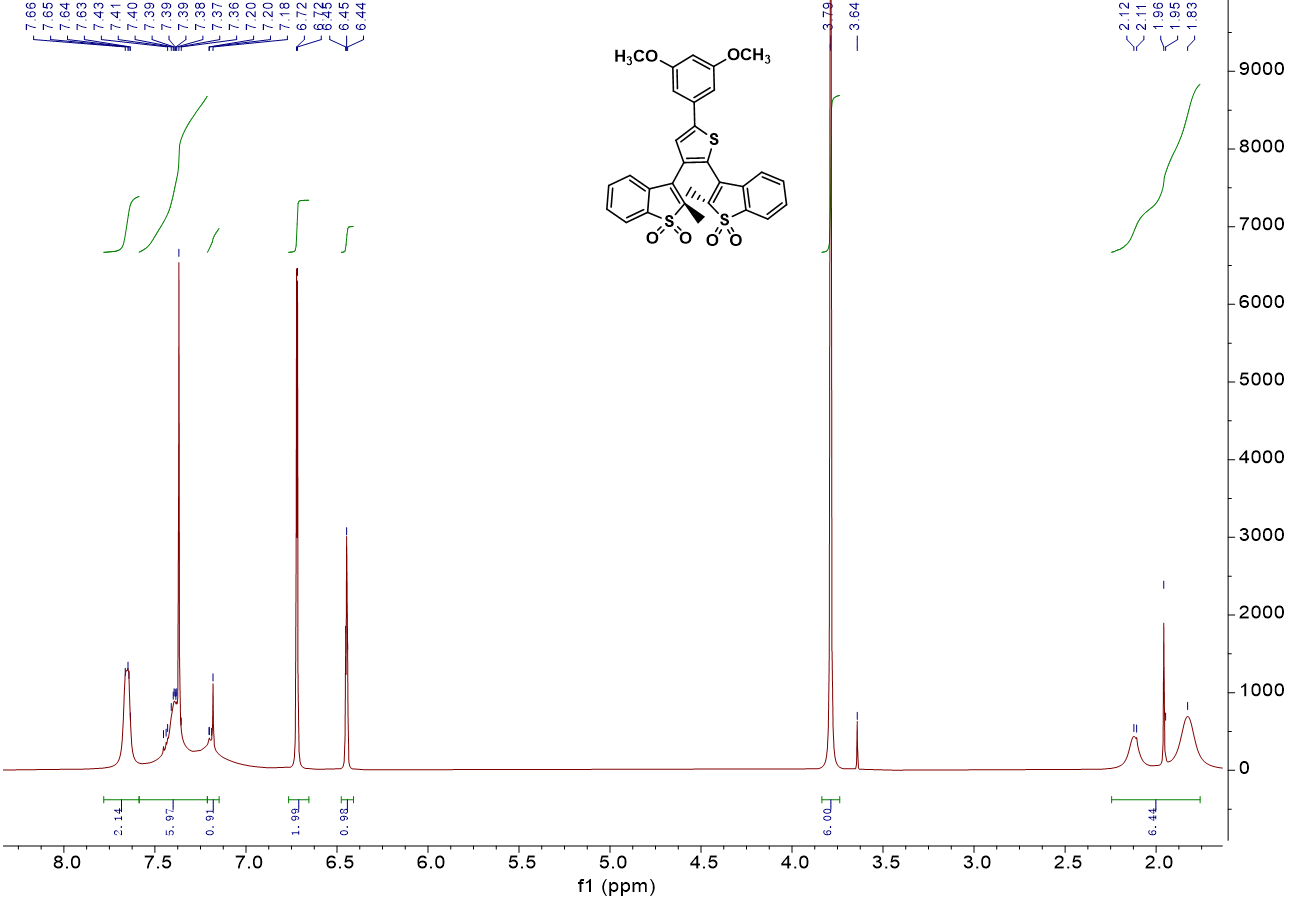


**Figure S4.** ^1^H NMR spectra of *m***-DMPB**.


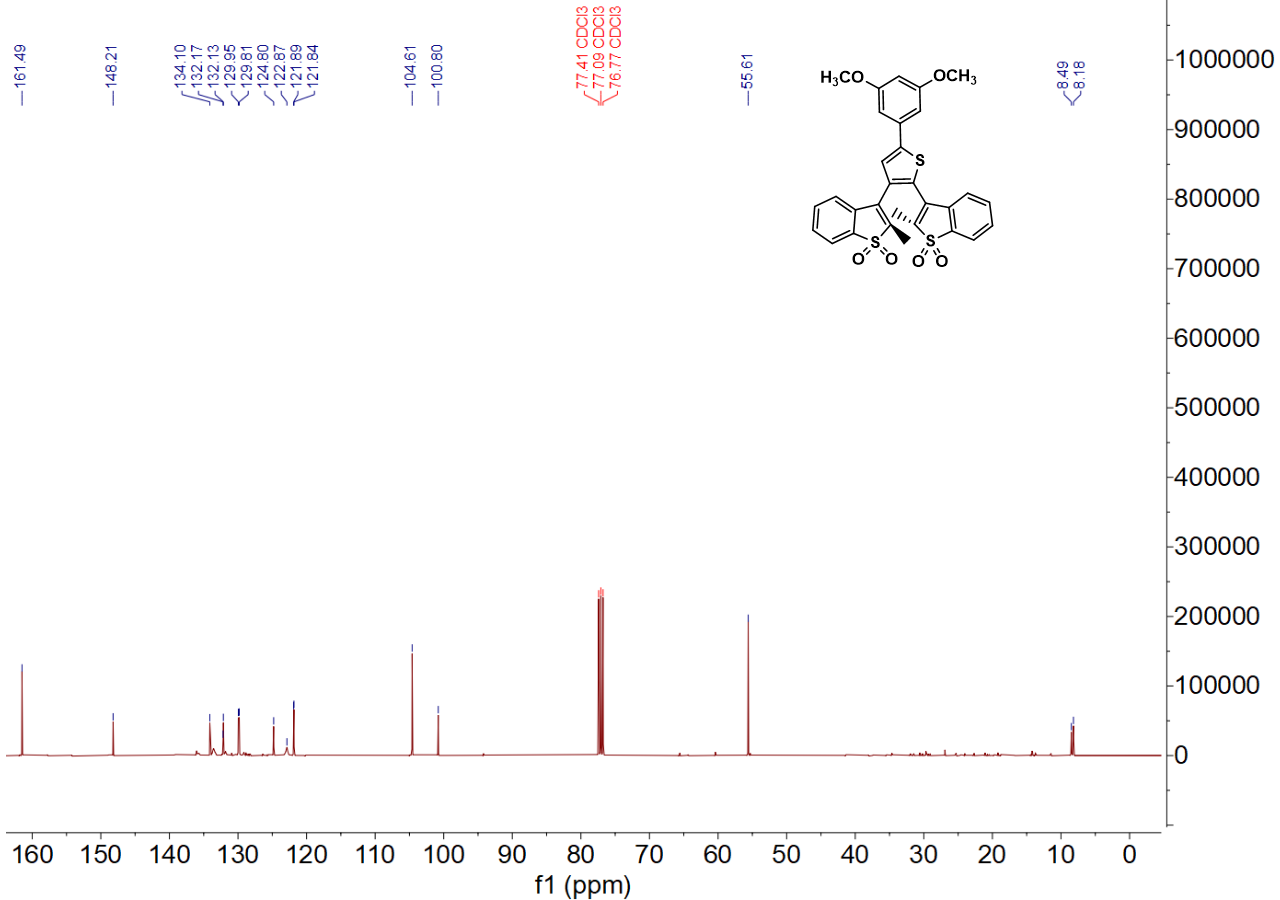


**Figure S5.** ^13^C NMR spectra of *m***-DMPB**.


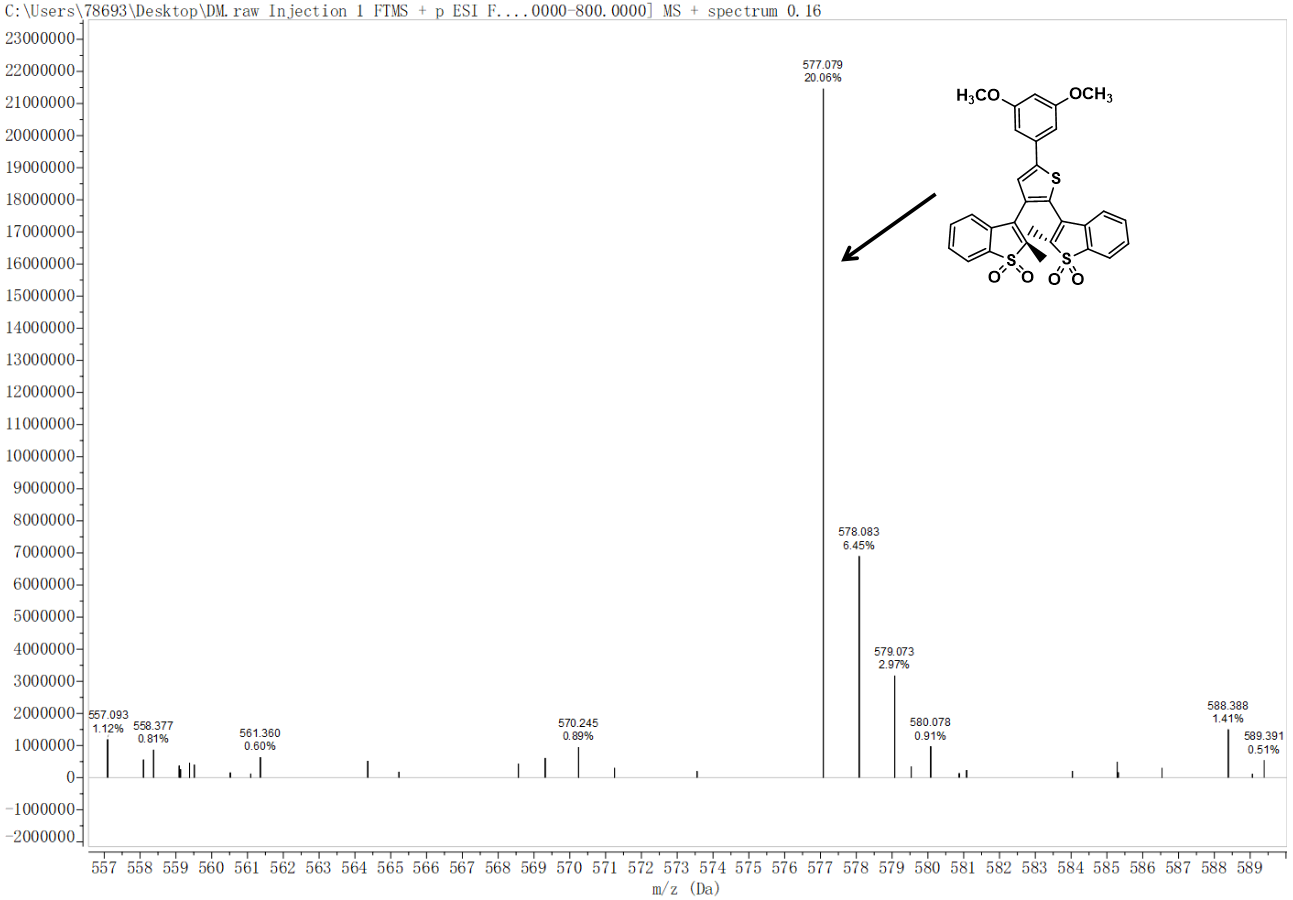


**Figure S6.** HRMS spectra of *m***-DMPB**.

**Figure S7.** ^1^H NMR spectra of *p***-DMPB**.

**Figure S8.** ^13^C NMR spectra of *p***-DMPB**.


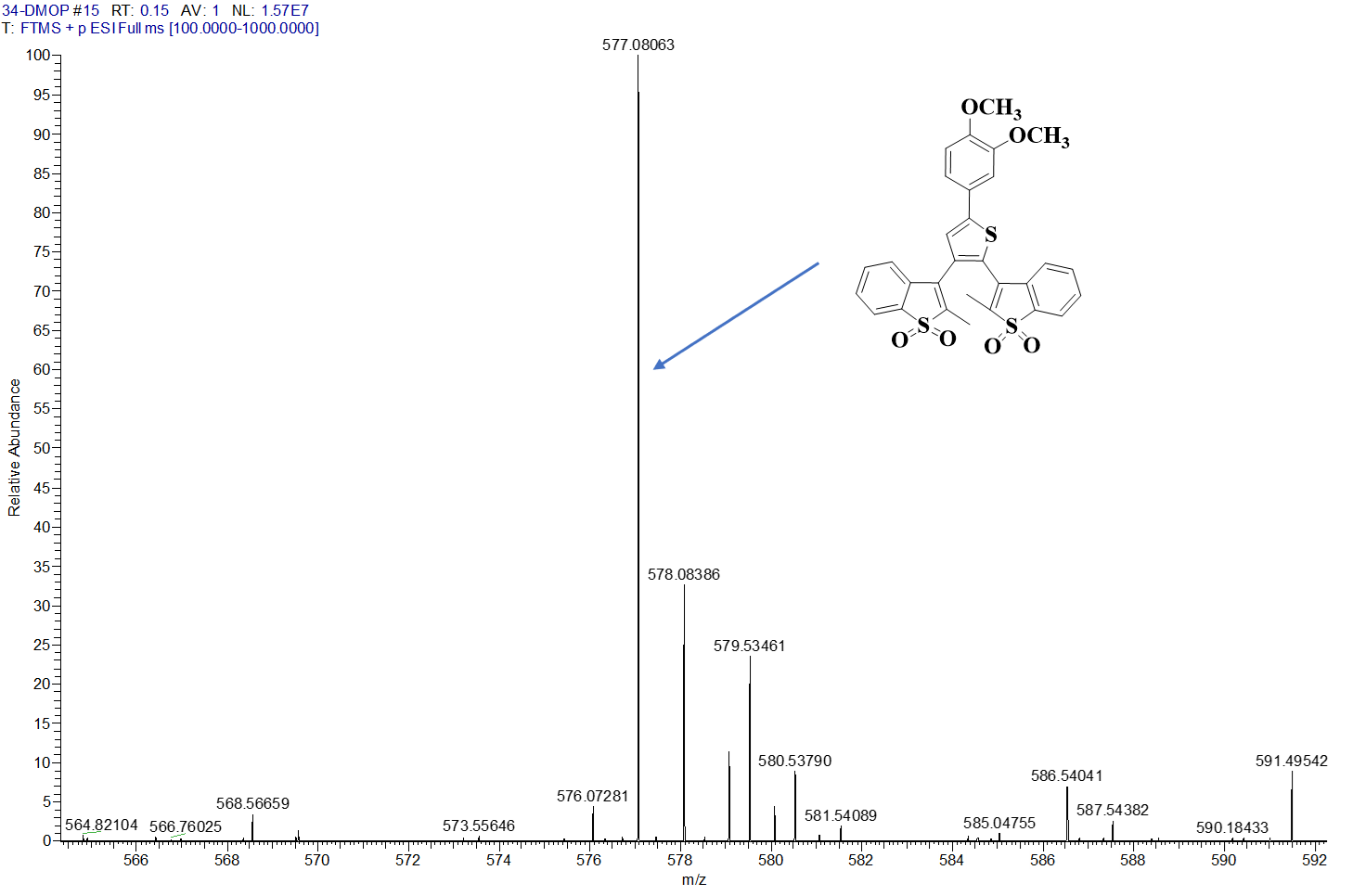


**Figure S9.** HRMS spectra of *p***-DMPB**.

4. Photophysical properties

The photo response time to achieve the photostationary state for these photoswitches were investigated in different solvents. As shown in **Figures** **S10**-**S12**, the photo response time to achieve the photostationary state for *o*-**DMPB** and *m*-**DMPB** with increasing solvent polarity, progressing from toluene to ethanol. This indicates that solvent polarity significantly influences the reaction kinetics of the cyclization process, where lower polarity corresponds to faster cyclization rates. However, for *p*-**DMPB**, the solvent polarity has a negligible effect on the photo response time. In summary, the choice of solvent, specifically toluene in this study, is appropriate for investigating the photochromic properties of these compounds.

The trends of light response times for both absorption and fluorescence spectra are consistent. However, the light response times for the ring-opening process differ from those for cyclization, typically requiring longer durations. The light response times for both the cyclization and cycloreversion processes are separated provided in **Figures** **S13**-**S15**.


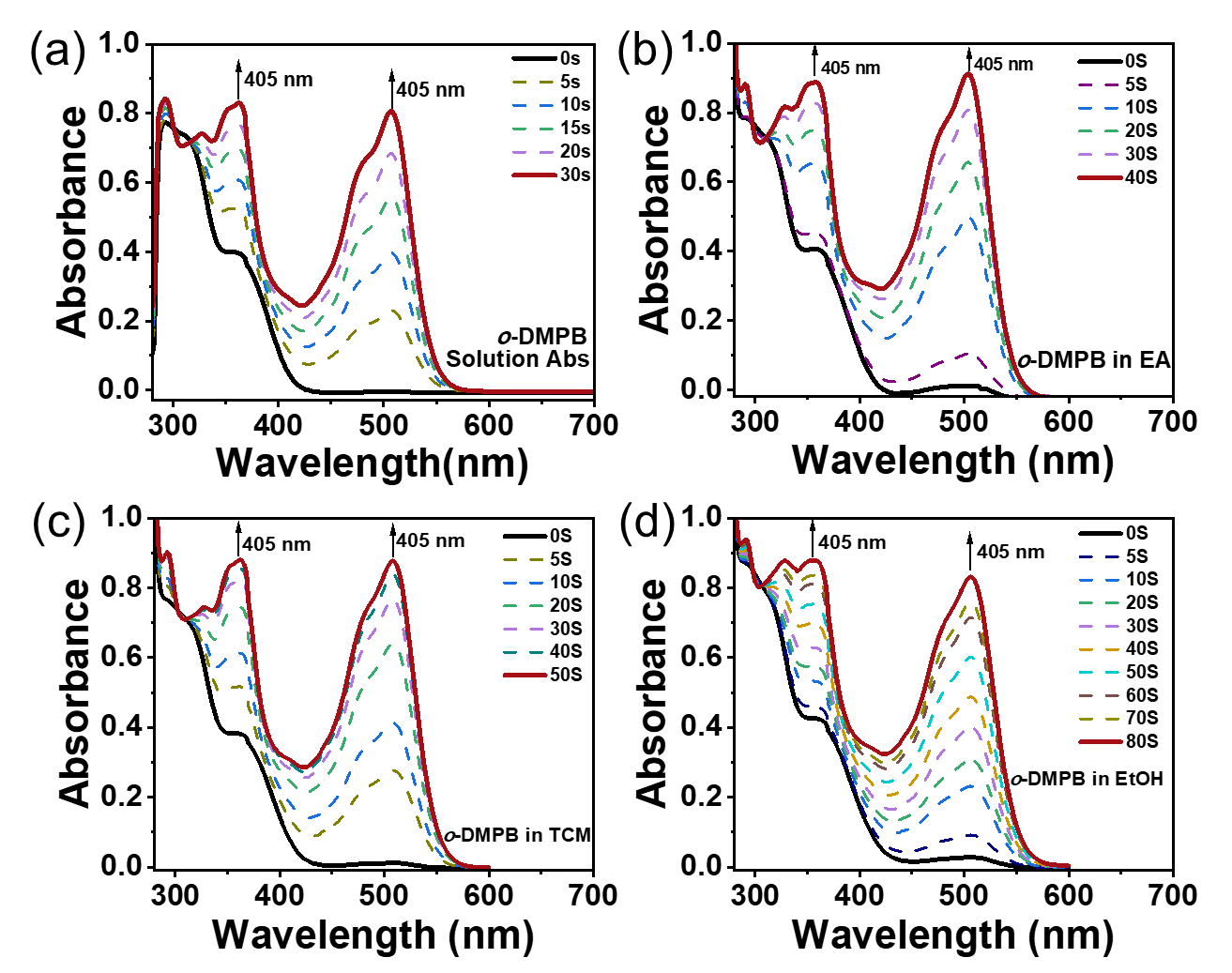


**Figure S10**. Changes in the absorption spectra of *o*-**DMPB** in toluene (a), ethyl acetate (b), chloroform (c), and ethanol (d) upon irradiation with visible light at 405 nm for different times. The concentration of all solutions is 5.0×10^-5^ M.


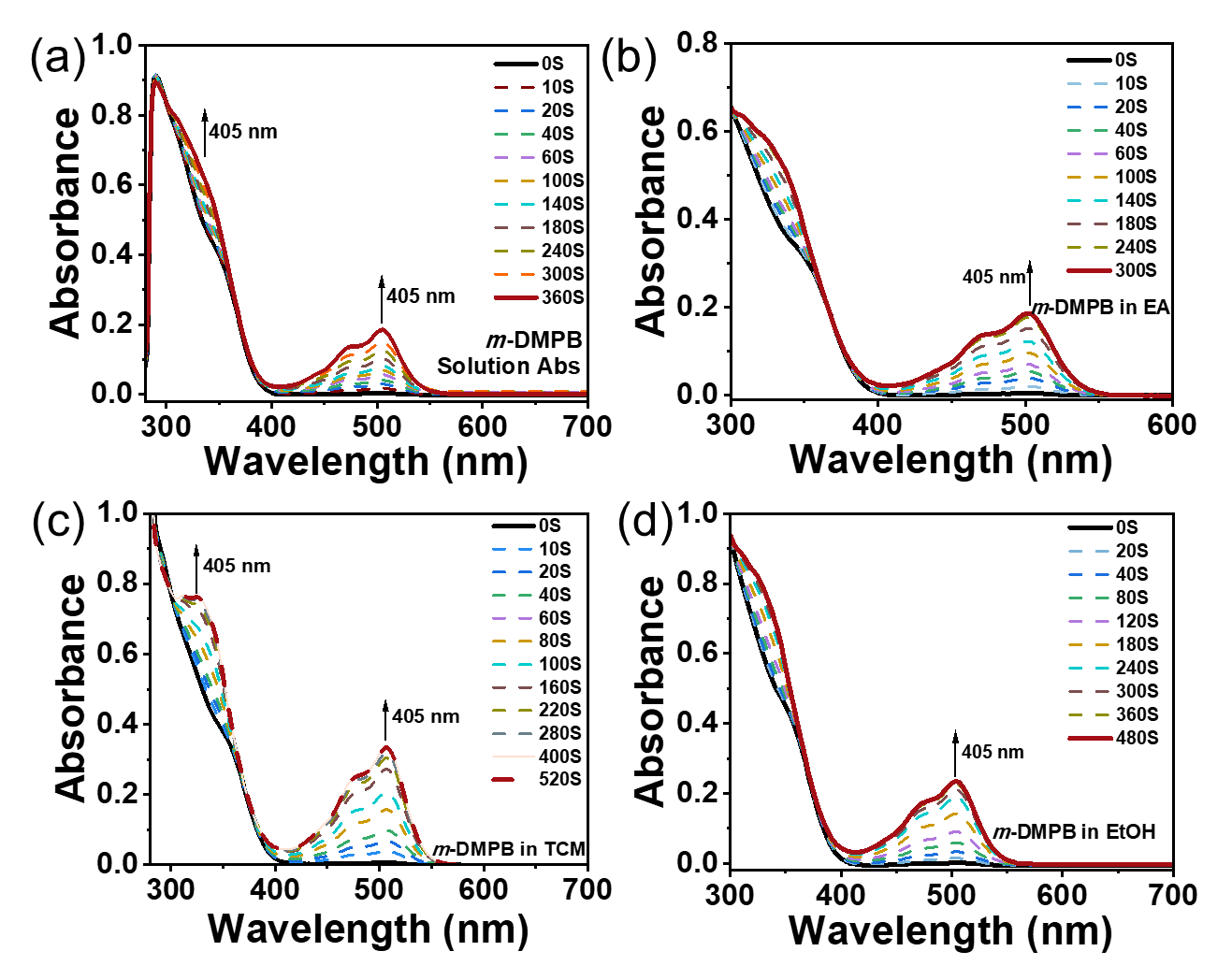


**Figure S11**. Changes in the absorption spectra of *m*-**DMPB** in toluene (a), ethyl acetate (b), chloroform (c), and ethanol (d) upon irradiation with visible light at 405 nm for different times. The concentration of all solutions is 5.0×10^-5^ M.


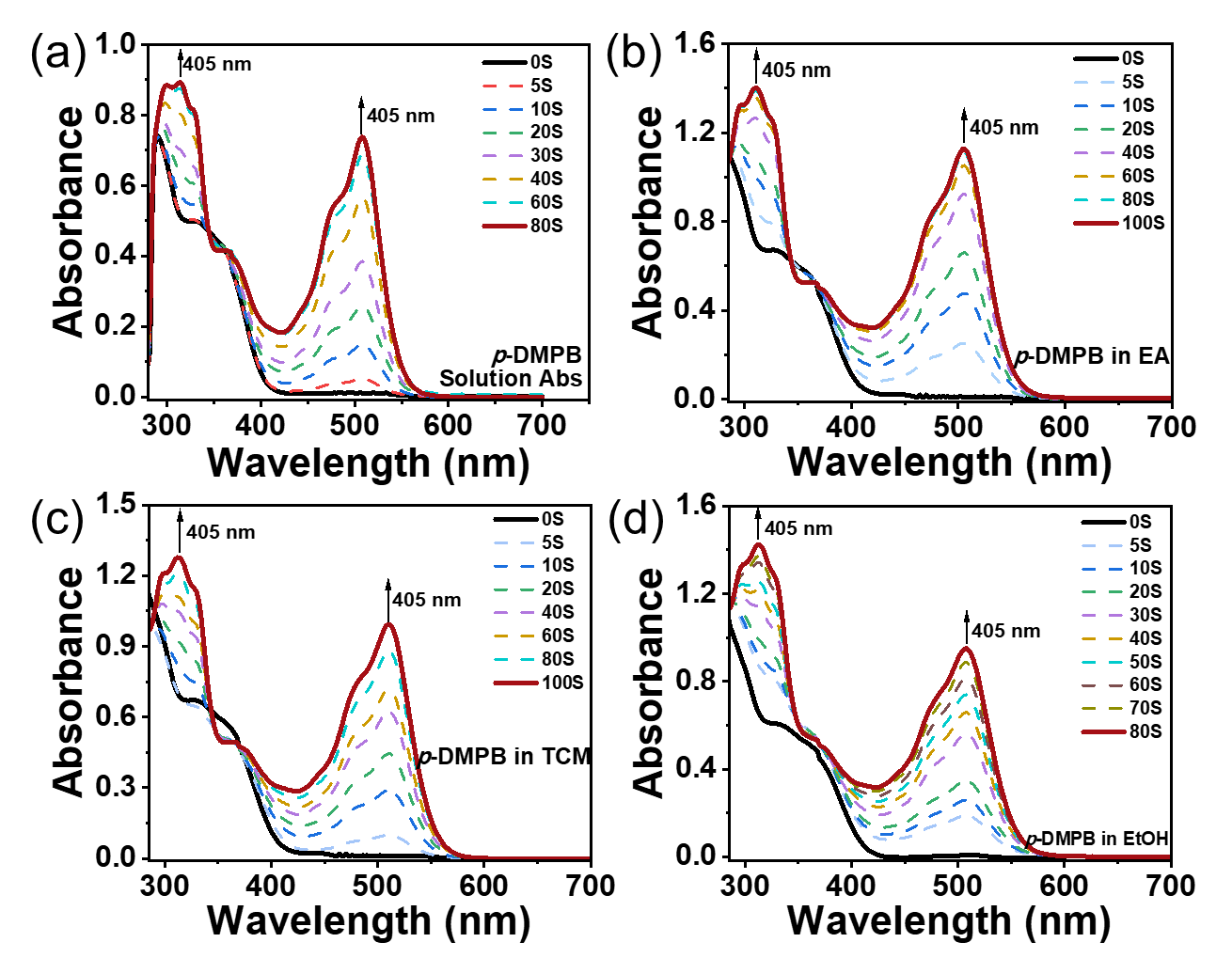


**Figure S12**. Changes in the absorption spectra of *p*-**DMPB** in toluene (a), ethyl acetate (b), chloroform (c), and ethanol (d) upon irradiation with visible light at 405 nm for different times. The concentration of all solutions is 5.0×10^-5^ M.


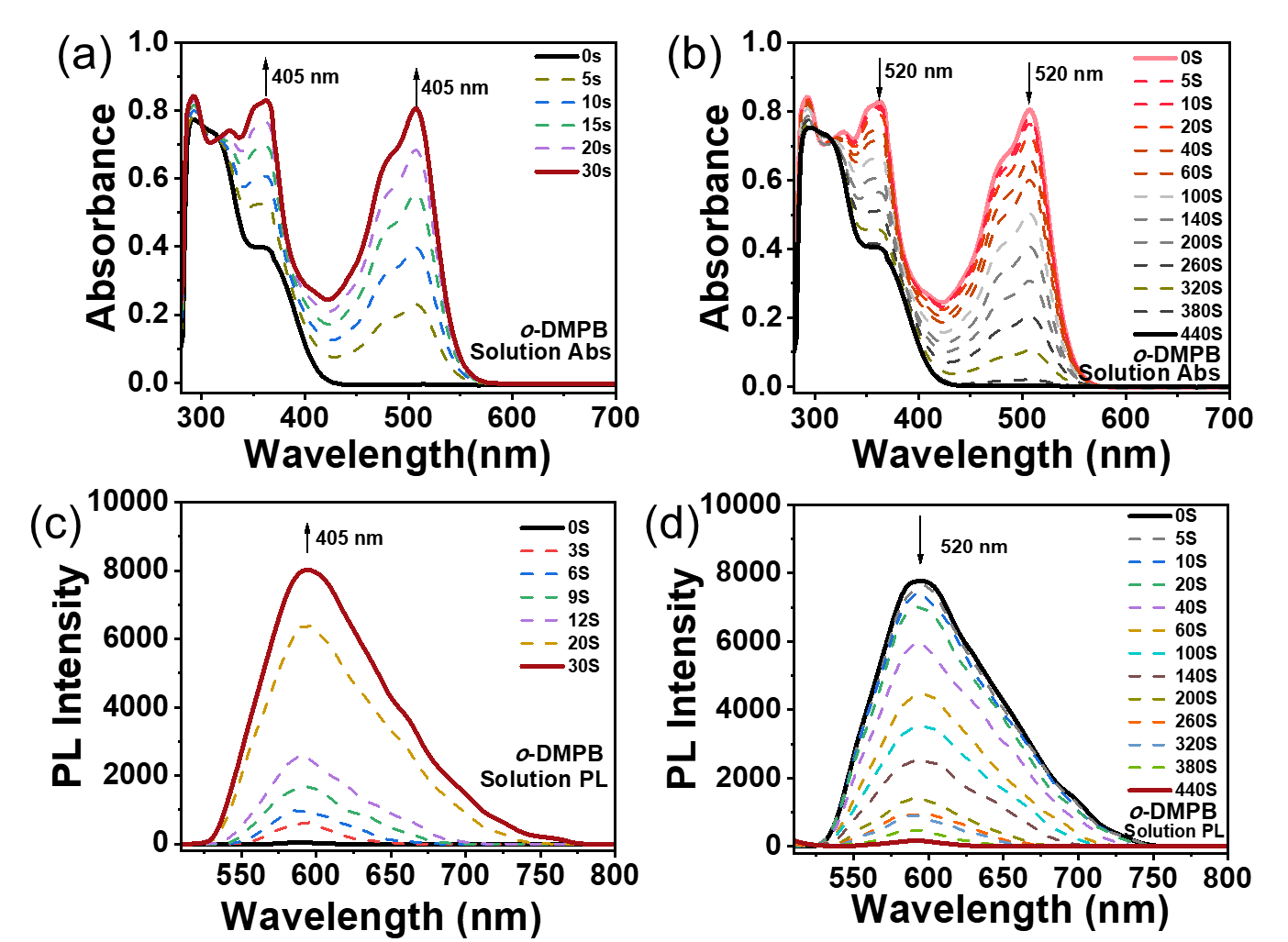


**Figure S13**. Changes in the absorption spectra of open form (a) and photostationary state (b) *o*-**DMPB** in toluene (5.0×10^-5^ M) upon continuing irradiation with visible light at 405 nm and 520 nm, respectively. Changes in the fluorescence spectra of open form (c) and photostationary state (d) *o*-**DMPB** in toluene (5.0×10^-5^ M) upon continuing irradiation with visible light at 405 nm and 520 nm, respectively. Excited at 405 nm, slit widths: 2.5 nm and 2.5 nm.


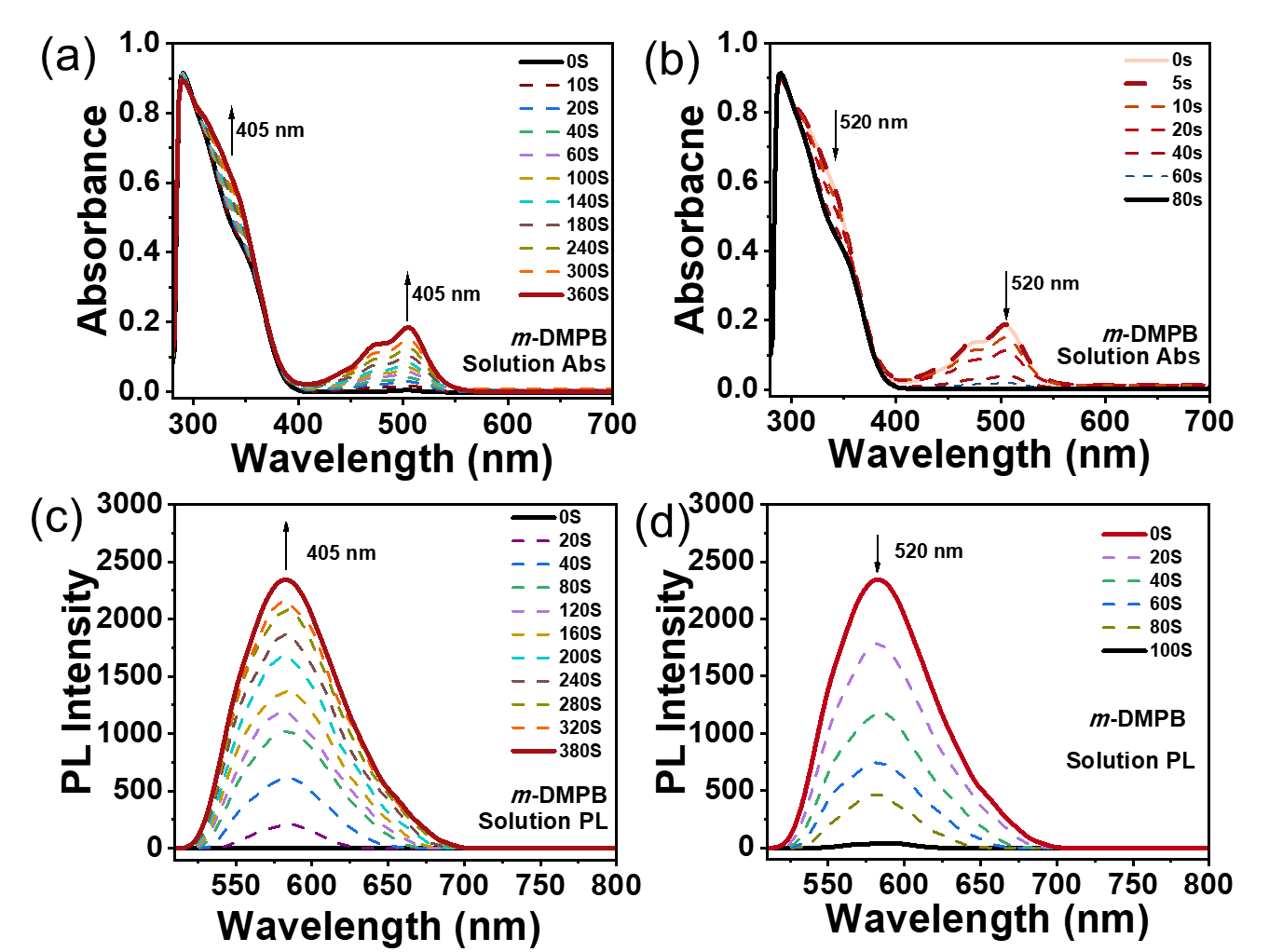


**Figure S14**. Changes in the absorption spectra of open form (a) and photostationary state (b) *m*-**DMPB** in toluene (5.0×10^-5^ M) upon continuing irradiation with visible light at 405 nm and 520 nm, respectively. Changes in the fluorescence spectra of open form (c) and photostationary state (d) *m*-**DMPB** in toluene (5.0×10^-5^ M) upon continuing irradiation with visible light at 405 nm and 520 nm, respectively. Excited at 405 nm, slit widths: 2.5 nm and 2.5 nm.


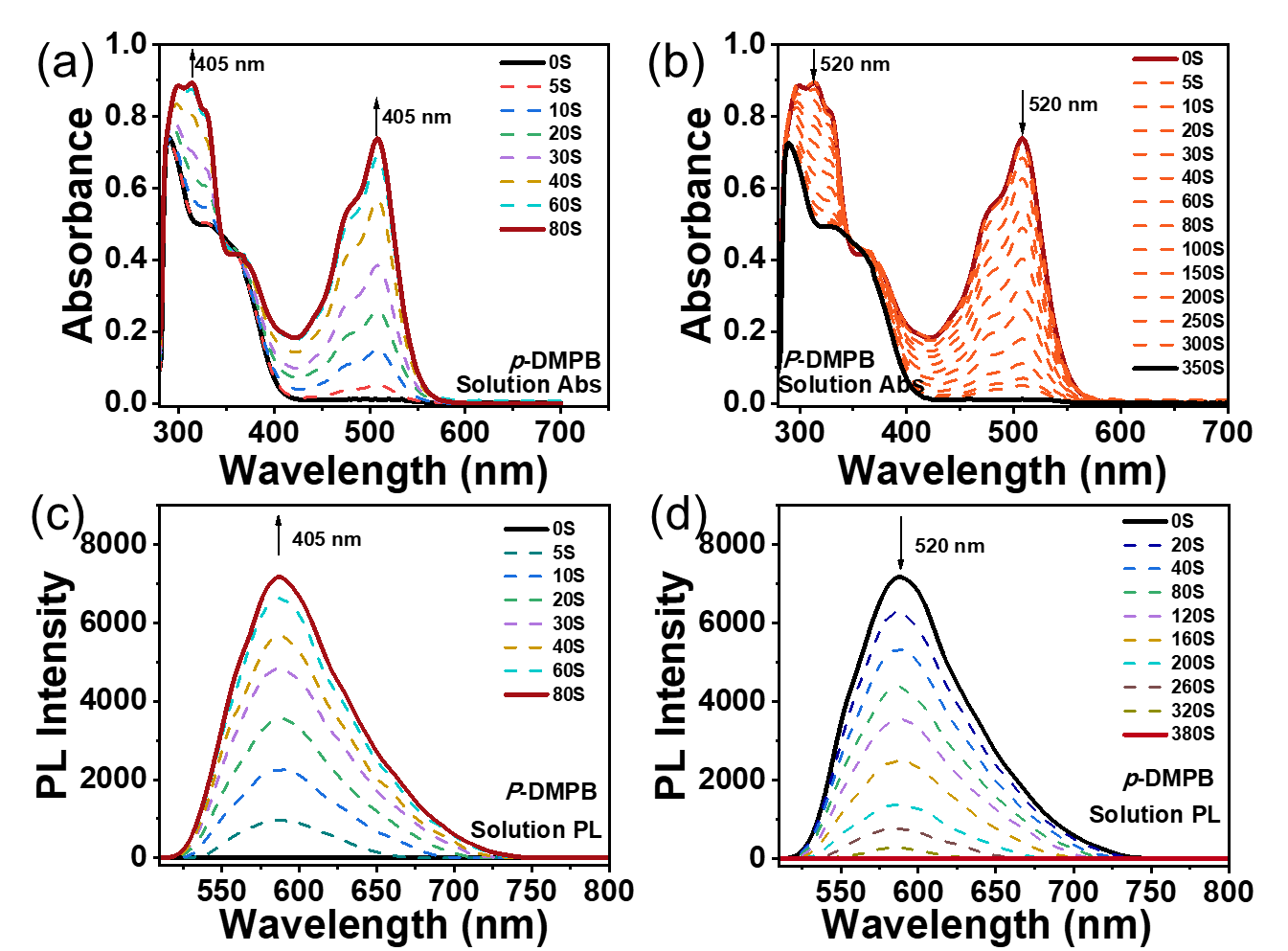


**Figure S15**. Changes in the absorption spectra of open form (a) and photostationary state (b) *p*-**DMPB** in toluene (5.0×10^-5^ M) upon continuing irradiation with visible light at 405 nm and 520 nm, respectively. Changes in the fluorescence spectra of open form (c) and photostationary state (d) *p*-**DMPB** in toluene (5.0×10^-5^ M) upon continuing irradiation with visible light at 405 nm and 520 nm, respectively. Excited at 405 nm, slit widths: 2.5 nm and 2.5 nm.

**Preparation of PMMA film doped with DMPB**

The solution of PMMA in chloroform (0.1 g/mL) was prepared by dissolving 1 g PMMA powder in 10 mL chloroform in a 20 mL sample bottle. Then 0.5 mL of the above solution was transferred to another bottle, followed by the addition of 1 mg **DMPB**. After ultrasonic dispersion, the **DMPB** was dissoved completely to give a solution of **DMPB** in the PMMA-chloroform mixture with a concentration of 2 mg/mL. For each film, the resulting **DMPB** solution (200 μL) was rapidly dropped on a clean quartz plate (2 cm × 2 cm), and then spincoated at 400 rpm for 5 s followed by 3000 rpm for 40 s to give a PMMA polymer film doped with 2 wt% **DMPB**.


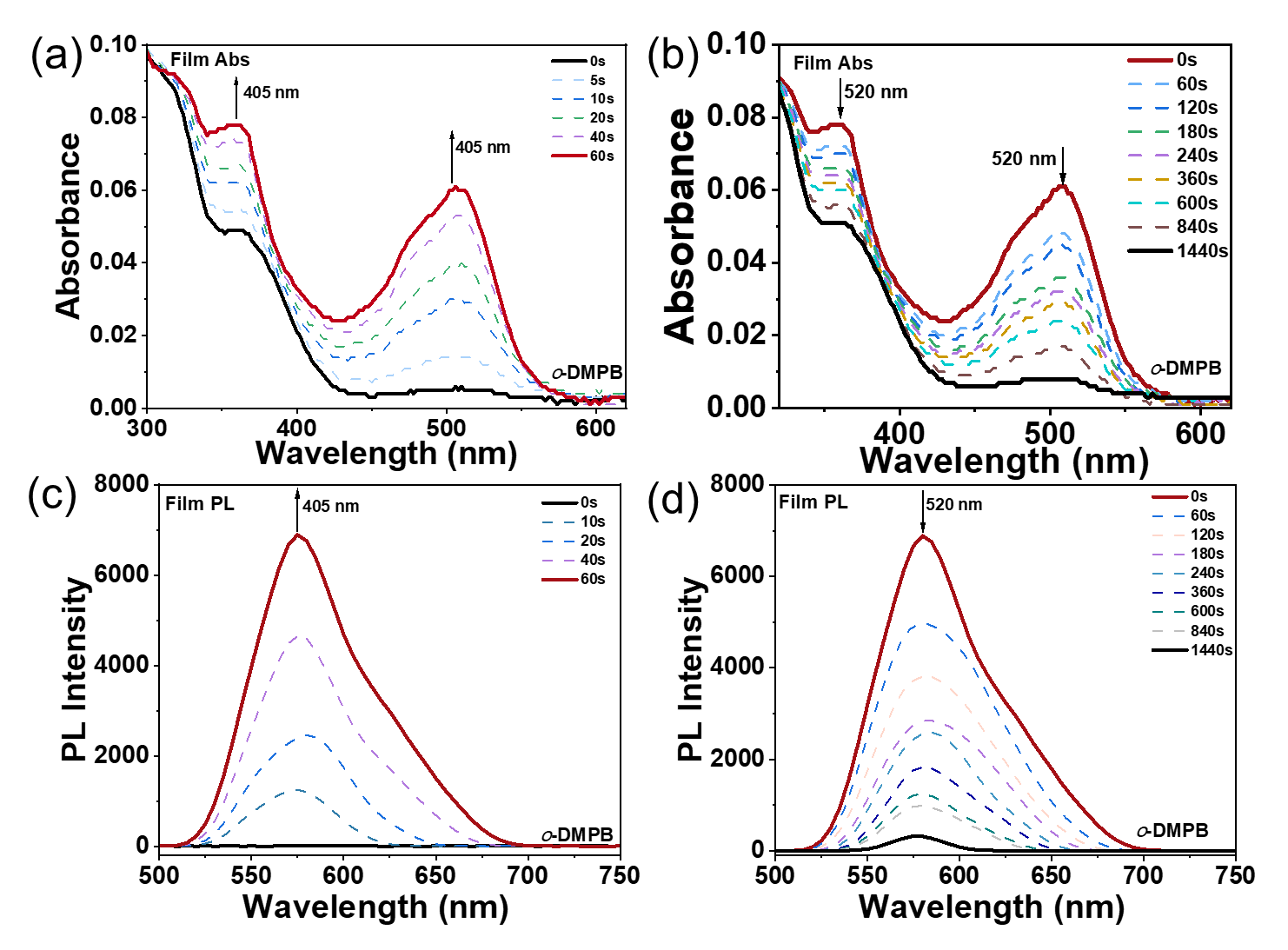


**Figure S16**. Changes in the absorption spectra of open form (a) and photostationary state (b) *o*-**DMPB** in film state upon continuing irradiation with visible light at 405 nm and 520 nm, respectively. Changes in the fluorescence spectra of open form (c) and photostationary state (d) *o*-**DMPB** in film state upon continuing irradiation with visible light at 405 nm and 520 nm, respectively. Excited at 405 nm, slit widths: 2.5 nm and 2.5 nm. The distance between the sample and the light source is 10 cm.

**
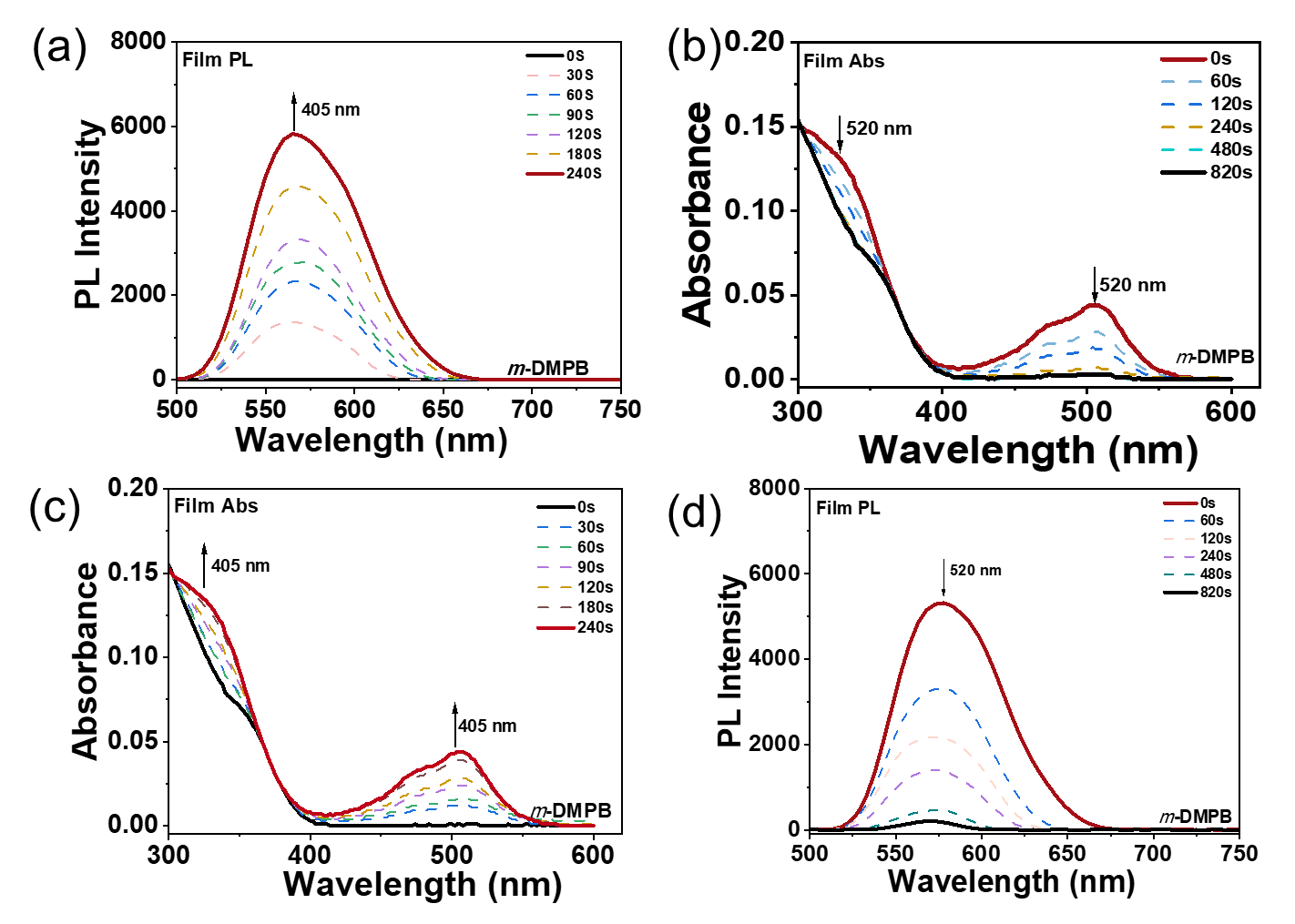
**

**Figure S17**. Changes in the absorption spectra of open form (a) and photostationary state (b) *m*-**DMPB** in film state upon continuing irradiation with visible light at 405 nm and 520 nm, respectively. Changes in the fluorescence spectra of open form (c) and photostationary state (d) *m*-**DMPB** in film state upon continuing irradiation with visible light at 405 nm and 520 nm, respectively. Excited at 405 nm, slit widths: 5 nm and 5 nm. The distance between the sample and the light source is 10 cm.

**
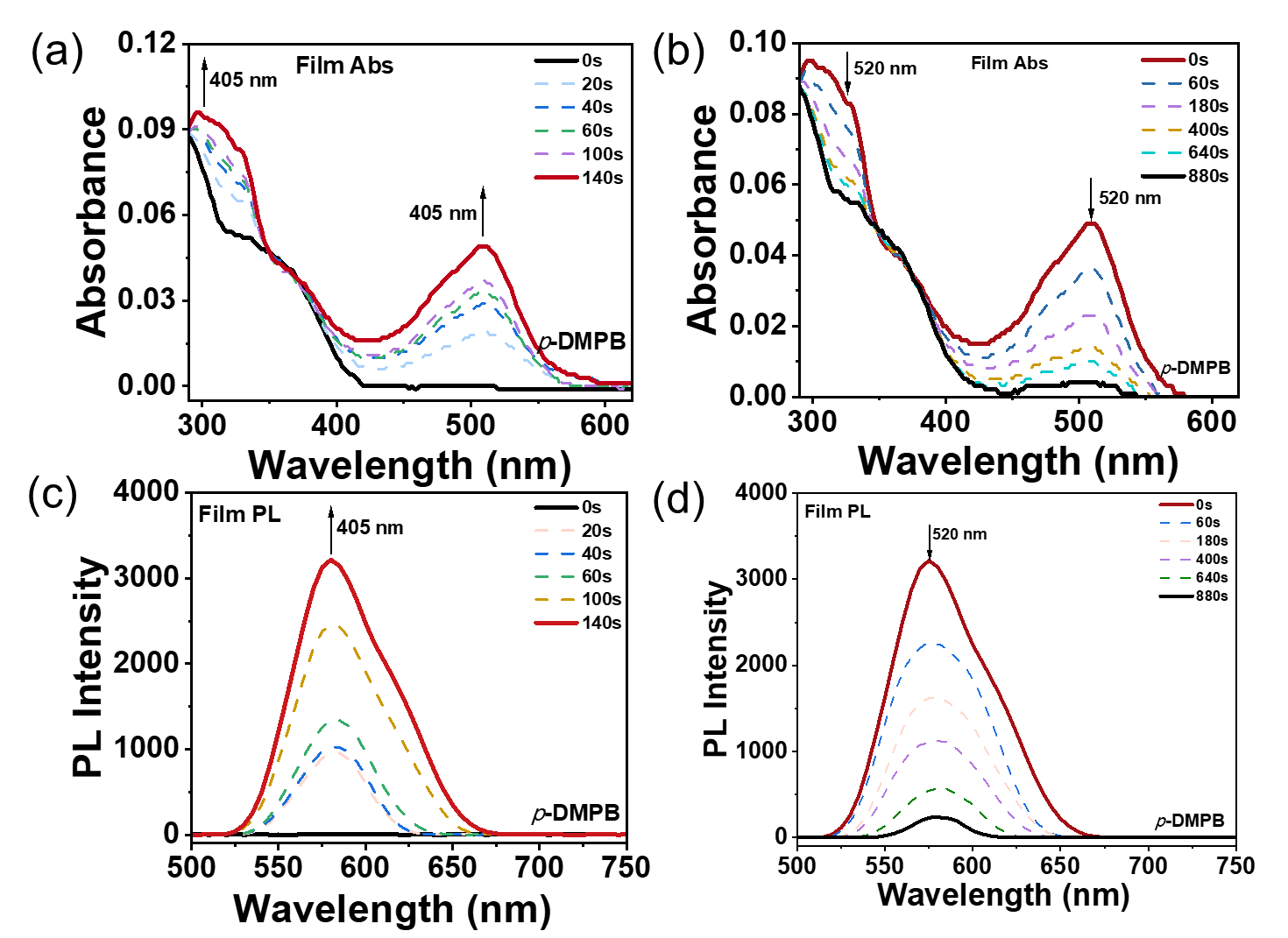
**

**Figure S18**. Changes in the absorption spectra of open form (a) and photostationary state (b) *p*-**DMPB** in film state upon continuing irradiation with visible light at 405 nm and 520 nm, respectively. Changes in the fluorescence spectra of open form (c) and photostationary state (d) *p*-**DMPB** in film state upon continuing irradiation with visible light at 405 nm and 520 nm, respectively. Excited at 405 nm, slit widths: 2.5 nm and 2.5 nm. The distance between the sample and the light source is 10 cm.

**Figure S19.** Absorption spectra of *m*-**DMPB**(c) in toluene (~ 5.0×10^-5^ M) before and after stored in the dark for 24 hours.

**Figure 20.** Absorbance switching of *m*-**DMPB** in toluene (~ 5.0×10^-5^ M) monitored at λ_max_ upon alternative irradiation with 365 nm and 520 nm.

**Figure S21.** The changes in the chemical shift of the methoxy groups in ^1^HNMR spectra (CDCl_3_) of *o*-**DMPB** before (down) and after (up) 365 nm light irradiation indicate a photocyclization reaction yield of 95%.

**Figure S22.** The changes in the chemical shift of the methoxy groups in ^1^HNMR spectra (CDCl_3_) of *o*-**DMPB** before (down) and after (up) 310 nm light irradiation indicate a photocyclization reaction yield of 60%.

**Figure S23.** The changes in the chemical shift of the methoxy groups in ^1^HNMR spectra (CDCl_3_) of *m*-**DMPB** before (down) and after (up) 365 nm light irradiation indicate a photocyclization reaction yield of 94%.

**Figure S24.** The changes in the chemical shift of the methoxy groups in ^1^HNMR spectra (CDCl_3_) of *m*-**DMPB** before (down) and after (up) 310 nm light irradiation indicate a photocyclization reaction yield of 73%.

**Figure S25.** The changes in the chemical shift of the methoxy groups in ^1^HNMR spectra (CDCl_3_) of *p*-**DMPB** before (down) and after (up) 365 nm light irradiation indicate a photocyclization reaction yield of 97%.

**Figure S26.** The changes in the chemical shift of the methoxy groups in ^1^HNMR spectra (CDCl_3_) of *p*-**DMPB** before (down) and after (up) 310 nm light irradiation indicate a photocyclization reaction yield of 82%.

**Figure S27.** Photochromism of BTF6 (a), *o*-**DMPB** (b), *m*-**DMPB** (c), and *p*-**DMPB** (d) (absorbance at 520 nm) in EA (1.0×10^-5^ M) upon irradiation with 310 and 520 nm light.

**Table S2**. Quantum yields of BTF6, *o*-**DMPB**, *m*-**DMPB,** and *p*-**DMPB** in EA (1.0×10^−5^ M).

| Compounds | Conversion at PSS (%)^a^ | Φ_o-c_ (310 nm) | Φ_c-o_ (520 nm) |
| --- | --- | --- | --- |
| BTF6 | 43.00 | 0.31 | 0.28 |
| *o*-**DMPB** | 60 | 0.35 | 0.00085 |
| *m*-**DMPB** | 73 | 0.17 | 0.0003 |
| *p*-**DMPB** | 82 | 0.44 | 0.0024 |

a: the conversion yield is obtained from **Figure S22**, **Figure S24**, and **Figure S26**.

5. Theoretical calculation

All calculations were performed using the Gaussian 09 program package^1^. The geometry of the ground state structures of *o*-**DMPB**, *m*-**DMPB**, and *p*-**DMPB** were optimized at the B3LYP/6-31g(d) level (Coordinates for geometries see **Appendix**). Only photochemically active anti-parallel conformation of the open-ring forms were considered.


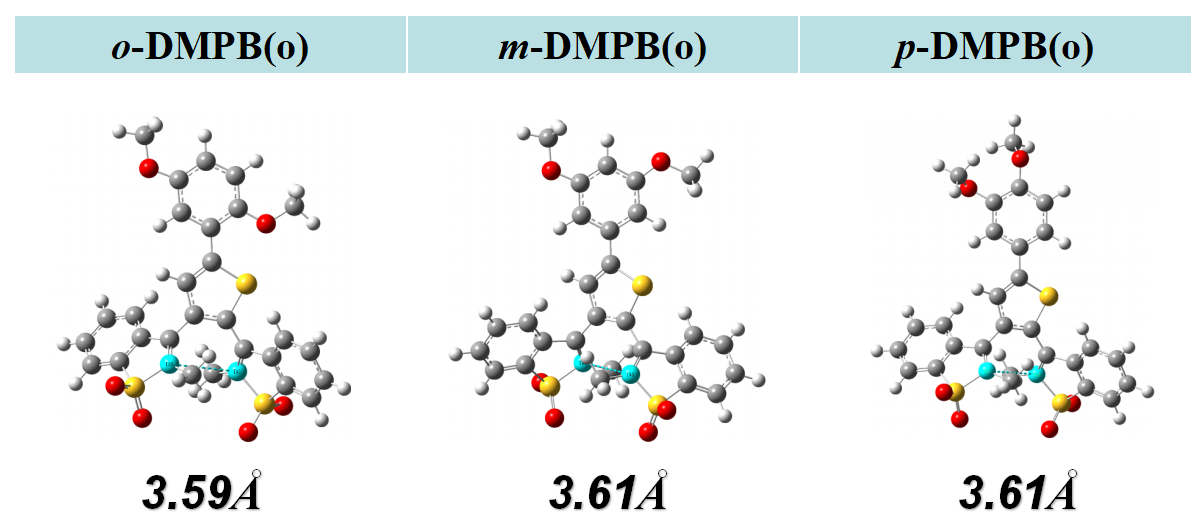


**Figure S28.** The calculated distance between the two active carbon atoms of *o*-**DMPB**(o), *m*-**DMPB**(o), and *p*-**DMPB**(o).

**Table S3**. Computed vertical absorption (S_VA_), oscillator strength (*f*), highest occupied molecular orbital energies (*E*_HOMO_), and lowest unoccupied molecular orbital energies (*E*_LUMO_).

| Parameter | *o*-**DMPB** | | *m*-**DMPB** | | *p*-**DMPB** | |  |
| --- | --- | --- | --- | --- | --- | --- | --- |
|  | Open | Close | Open | Close | Open | Close | |
| S_VA_ (eV)^a^ | 3.19 | 2.42 | 3.36 | 2.47 | 3.34 | 2.47 | |
| *f* ^a^ | - | 0.3374 | - | 0.3474 | - | 0.3524 | |
| *E*_HOMO_(eV) ^b^ | -6.20 | -5.30 | -6.06 | -5.44 | -6.06 | -5.45 | |
| *E*_LUMO_(eV) ^b^ | -2.11 | -2.62 | -2.24 | -2.72 | -2.26 | -2.74 | |
| *E*_gap_(eV) | 3.64 | 2.68 | 3.82 | 2.72 | 3.80 | 2.71 | |

a Calculated by TD-DFT/B3LYP/6-31G(d).

b Derived from DFT/ B3LYP/6-31G(d).

**
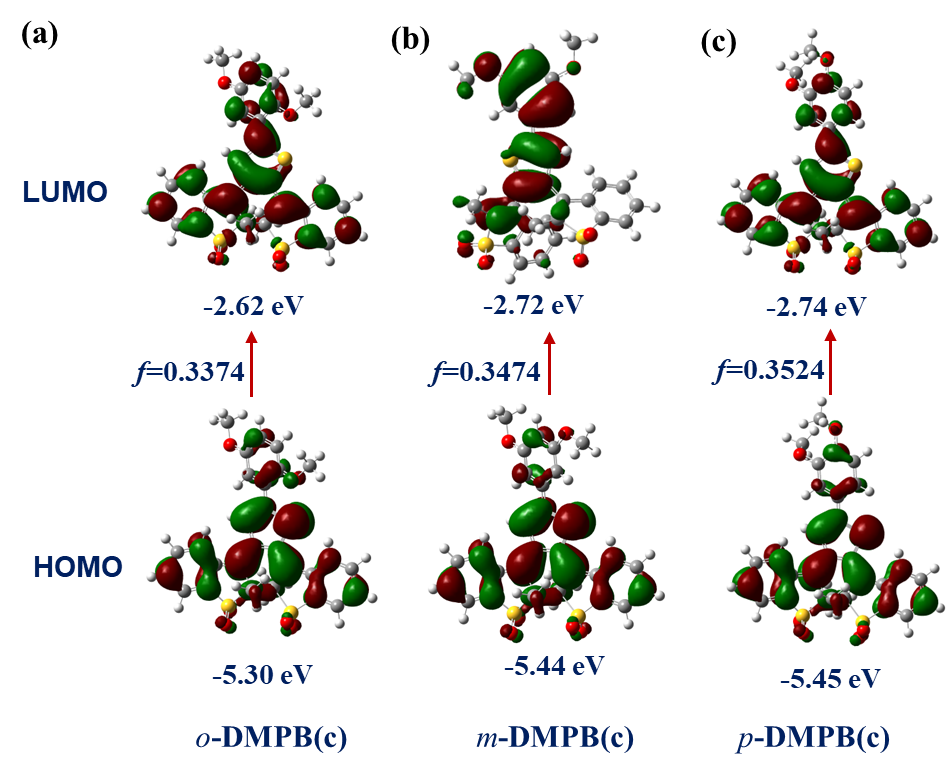
**

**Figure S29.** The highest occupied and lowest unoccupied molecular orbitals (HOMO and LUMO) of the closed-ring forms of *o*-**DMPB** (a), *m*-**DMPB** (b), and *p*-**DMPB** (c).

6. References

1. Frisch, M. J.; Trucks, G. W.; Schlegel, H. B.; Scuseria, G. E.; Robb, M. A.; Cheeseman, J. R.; Scalmani, G.; Barone, V.; Mennucci, B.; Petersson, G. A.; Nakatsuji, H.; Caricato, M.; Li, X.; Hratchian, H. P.; Izmaylov, A. F.; Bloino, J.; Zheng, G.; Sonnenberg, J. L.; Hada, M.; Ehara, M.; Toyota, K.; Fukuda, R.; Hasegawa, J.; Ishida, M.; Nakajima, T.; Honda, Y.; Kitao, O.; Nakai, H.; Vreven, T.; Montgomery, J. A.; Peralta, J. E.; Ogliaro, F.; Bearpark, M.; Heyd, J. J.; Brothers, E.; Kudin, K. N.; Staroverov, V. N.; Keith, T.; Kobayashi, R.; Normand, J.; Raghavachari, K.; Rendell, A.; Burant, J. C., Iyengar, S. S.; Tomasi, J.; Cossi, M.; Rega, N.; Millam, J. M.; Klene, M.; Knox, J. E.; Cross, J. B.; Bakken, V.; Adamo, C.; Jaramillo, J.; Gomperts, R.; Stratmann, R. E.; Yazyev, O.; Austin, A. J.; Cammi, R.; Pomelli, C.; Ochterski, J. W.; Martin, R. L.; Morokuma, K.; Zakrzewski, V. G.; Voth, G. A.; Salvador, P.; Dannenberg, J. J.; Dapprich, S.; Daniels, A. D.; Farkas, Ö.; Foresman, J. B.; Ortiz, J. V.; Cioslowski, J.; Fox, D. J. Gaussian Inc.,Wallingford CT, **2009**.

7. Appendix

Coordinates for calculated geometries:

*o***-DMPB**(o) optimized at the B3LYP/6-31G(d) level.

| Atom/ Å | X/ Å | Y/ Å | Z/ Å |
| --- | --- | --- | --- |
| C | -1.01256 | -4.688 | 1.3077 |
| C | -2.24757 | -4.9848 | 0.88153 |
| C | -2.92699 | -4.06671 | 0.18195 |
| C | -2.34564 | -2.8925 | -0.10838 |
| C | -1.09074 | -2.58243 | 0.2591 |
| C | -0.45393 | -3.50239 | 1.01203 |
| S | -3.29717 | -1.60641 | -0.92368 |
| C | -1.70984 | -0.76251 | -0.82792 |
| C | -0.6937 | -1.35409 | -0.15565 |
| C | 0.51881 | -0.77131 | 0.01767 |
| C | 0.86453 | 0.53142 | 0.06738 |
| C | 2.82927 | -0.57306 | 0.09421 |
| C | 0.09874 | 1.63685 | 0.18083 |
| C | -0.97375 | 1.71505 | 0.99784 |
| S | -1.83223 | 3.29493 | 1.09161 |
| C | -0.51112 | 3.78867 | -0.02218 |
| C | 0.34651 | 2.82843 | -0.39889 |
| C | -0.43108 | 5.0384 | -0.50354 |
| C | 0.51717 | 5.3385 | -1.40305 |
| C | 1.36177 | 4.38257 | -1.81792 |
| C | 1.27164 | 3.13578 | -1.32682 |
| C | -1.59267 | 0.41305 | -1.77461 |
| C | -1.41102 | 0.67322 | 2.00123 |
| O | -4.24196 | -0.99896 | 0.00318 |
| O | -3.61545 | -1.99381 | -2.29086 |
| O | -3.09769 | 3.22889 | 0.37394 |
| O | -1.65425 | 3.91484 | 2.39729 |
| C | 4.15747 | -0.87009 | 0.10247 |
| C | 4.55703 | -2.15581 | 0.25621 |
| C | 5.13839 | 0.06319 | -0.03764 |
| C | 5.82776 | -2.59845 | 0.28958 |
| C | 6.4162 | -0.36872 | -0.01933 |
| C | 6.76525 | -1.65016 | 0.14554 |
| C | 2.20454 | 0.61447 | 0.07556 |
| S | 1.74433 | -1.55749 | 0.19262 |
| O | 6.07139 | -3.9413 | 0.4597 |
| O | 4.83768 | 1.38828 | -0.24357 |
| C | 7.40684 | -4.38898 | 0.4925 |
| C | 5.8409 | 2.303 | -0.61842 |
| H | -0.46189 | -5.42162 | 1.92142 |
| H | -2.71175 | -5.95171 | 1.13664 |
| H | -3.96314 | -4.27893 | -0.12977 |
| H | 0.54278 | -3.3473 | 1.44515 |
| H | -1.13738 | 5.8168 | -0.17103 |
| H | 0.58943 | 6.35955 | -1.81316 |
| H | 2.12162 | 4.6201 | -2.58175 |
| H | 1.95647 | 2.38221 | -1.74539 |
| H | -2.09771 | 0.18766 | -2.74056 |
| H | -2.09635 | 1.31766 | -1.37166 |
| H | -0.54921 | 0.64004 | -2.07879 |
| H | -1.63604 | 1.14707 | 2.98309 |
| H | -2.34141 | 0.16555 | 1.66531 |
| H | -0.63259 | -0.08667 | 2.22433 |
| H | 3.831 | -2.97739 | 0.37865 |
| H | 7.25528 | 0.33447 | -0.14177 |
| H | 7.83869 | -1.89698 | 0.1511 |
| H | 2.70351 | 1.58508 | 0.14147 |
| H | 7.39191 | -5.49245 | 0.63831 |
| H | 7.91118 | -4.17382 | -0.4759 |
| H | 7.94704 | -3.93274 | 1.35188 |
| H | 6.56887 | 2.44697 | 0.21058 |
| H | 6.33241 | 1.97657 | -1.56203 |
| H | 5.34691 | 3.28259 | -0.80866 |

*o***-DMPB**(o) optimized at the TD B3LYP/6-31G(d) level.

| Atom/ Å | X/ Å | Y/ Å | Z/ Å |
| --- | --- | --- | --- |
| C | 1.11611 | 4.85066 | 1.55209 |
| C | 2.31049 | 5.43673 | 1.13093 |
| C | 3.14111 | 4.76227 | 0.22733 |
| C | 2.72385 | 3.52611 | -0.23342 |
| C | 1.52498 | 2.91879 | 0.16157 |
| C | 0.71806 | 3.5938 | 1.07997 |
| S | 3.6172 | 2.42465 | -1.32476 |
| C | 2.3103 | 1.17902 | -1.27331 |
| C | 1.3058 | 1.57397 | -0.45709 |
| C | 0.09364 | 0.78699 | -0.18838 |
| C | -0.04613 | -0.53608 | 0.20817 |
| C | -2.32578 | 0.04633 | 0.00094 |
| C | 1.04932 | -1.48316 | 0.50659 |
| C | 2.08231 | -1.26254 | 1.34845 |
| S | 3.16776 | -2.70568 | 1.41605 |
| C | 2.13887 | -3.6361 | 0.28348 |
| C | 1.06191 | -2.83883 | -0.12334 |
| C | 2.36019 | -4.91771 | -0.18887 |
| C | 1.45038 | -5.43639 | -1.11887 |
| C | 0.37268 | -4.66068 | -1.54964 |
| C | 0.17092 | -3.36301 | -1.06327 |
| C | 2.50639 | -0.03093 | -2.11917 |
| C | 2.45406 | -0.10393 | 2.20857 |
| O | 4.82958 | 1.92229 | -0.65753 |
| O | 3.72152 | 2.97198 | -2.68706 |
| O | 4.4642 | -2.39866 | 0.78926 |
| O | 3.14351 | -3.28302 | 2.76999 |
| C | -3.79457 | 0.01516 | 0.01618 |
| C | -4.51877 | 1.19561 | 0.21503 |
| C | -4.52343 | -1.18893 | -0.18809 |
| C | -5.91727 | 1.22256 | 0.21186 |
| C | -5.91677 | -1.15887 | -0.18842 |
| C | -6.6198 | 0.03479 | 0.00911 |
| C | -1.41608 | -0.93503 | 0.31173 |
| S | -1.47953 | 1.51305 | -0.45307 |
| O | -6.48511 | 2.44747 | 0.42323 |
| O | -3.79267 | -2.32945 | -0.38596 |
| C | -7.90076 | 2.53114 | 0.43603 |
| C | -4.48529 | -3.54371 | -0.63155 |
| H | 0.48223 | 5.37342 | 2.26271 |
| H | 2.6024 | 6.41208 | 1.50852 |
| H | 4.08288 | 5.19129 | -0.10088 |
| H | -0.20882 | 3.15417 | 1.43093 |
| H | 3.21317 | -5.49819 | 0.14912 |
| H | 1.59034 | -6.44006 | -1.5091 |
| H | -0.32125 | -5.06591 | -2.28096 |
| H | -0.67138 | -2.77589 | -1.41404 |
| H | 2.84981 | 0.26654 | -3.11716 |
| H | 3.2732 | -0.68684 | -1.68713 |
| H | 1.57694 | -0.59669 | -2.21726 |
| H | 2.70788 | -0.4545 | 3.2161 |
| H | 3.33528 | 0.41176 | 1.80577 |
| H | 1.6318 | 0.6121 | 2.27886 |
| H | -3.99953 | 2.13087 | 0.39802 |
| H | -6.48196 | -2.06978 | -0.34525 |
| H | -7.70324 | 0.0128 | 0.00502 |
| H | -1.71605 | -1.9187 | 0.64272 |
| H | -8.1373 | 3.58124 | 0.61622 |
| H | -8.3327 | 2.22277 | -0.52561 |
| H | -8.33464 | 1.9194 | 1.23855 |
| H | -5.12088 | -3.82474 | 0.21817 |
| H | -5.10012 | -3.47987 | -1.53875 |
| H | -3.7134 | -4.30292 | -0.76959 |

*o***-DMPB**(c) optimized at the B3LYP/6-31G(d) level.

| Atom/ Å | X/ Å | Y/ Å | Z/ Å |
| --- | --- | --- | --- |
| C | -10.0708 | -1.18845 | 0.30193 |
| C | -10.39234 | -2.30777 | 0.96483 |
| C | -9.41663 | -3.04984 | 1.5064 |
| C | -8.13677 | -2.65347 | 1.40954 |
| C | -7.80235 | -1.509 | 0.7885 |
| C | -8.78798 | -0.80339 | 0.20513 |
| S | -6.84949 | -3.7242 | 2.06639 |
| C | -5.68997 | -2.37594 | 1.52198 |
| C | -6.47533 | -1.28977 | 0.8004 |
| C | -5.73544 | -0.27366 | 0.30849 |
| C | -4.40294 | -0.37284 | 0.1455 |
| S | -3.86816 | 0.90945 | -0.30467 |
| C | -5.0757 | 1.73455 | -0.47285 |
| C | -6.09925 | 0.95729 | -0.07468 |
| C | -3.72744 | -1.52888 | 0.32802 |
| C | -4.57902 | -2.77997 | 0.50786 |
| S | -3.48785 | -4.20558 | 1.00149 |
| C | -2.14073 | -3.0793 | 0.6127 |
| C | -2.40618 | -1.79189 | 0.32882 |
| C | -0.87997 | -3.5391 | 0.67216 |
| C | 0.14908 | -2.70822 | 0.45668 |
| C | -0.09717 | -1.41916 | 0.18623 |
| C | -1.36071 | -0.96815 | 0.1271 |
| C | -5.06908 | 3.024 | -0.91335 |
| O | -6.62884 | -4.8825 | 1.2218 |
| O | -3.52133 | -5.27021 | 0.00983 |
| O | -3.63326 | -4.40635 | 2.43059 |
| C | -6.19372 | 3.77198 | -1.08747 |
| C | -6.04102 | 5.03557 | -1.53486 |
| C | -4.85418 | 5.59112 | -1.80569 |
| C | -3.72307 | 4.88905 | -1.64534 |
| C | -3.89093 | 3.62809 | -1.2053 |
| C | -5.17691 | -1.75291 | 2.84749 |
| C | -5.09754 | -3.15346 | -0.9069 |
| O | -2.45339 | 5.35499 | -1.8955 |
| O | -7.44197 | 3.25224 | -0.8431 |
| C | -2.29579 | 6.67278 | -2.3683 |
| C | -8.6033 | 3.99291 | -1.137 |
| O | -6.98545 | -3.77109 | 3.51467 |
| H | -10.86547 | -0.59485 | -0.1815 |
| H | -11.44393 | -2.63213 | 1.03399 |
| H | -9.67938 | -3.992 | 2.01607 |
| H | -8.60637 | 0.0962 | -0.3942 |
| H | -7.13281 | 1.28427 | 0.00199 |
| H | -0.67506 | -4.59882 | 0.89904 |
| H | 1.1862 | -3.07937 | 0.50645 |
| H | 0.74625 | -0.72834 | 0.01583 |
| H | -1.47155 | 0.102 | -0.08979 |
| H | -6.91166 | 5.68909 | -1.70301 |
| H | -4.83948 | 6.63243 | -2.16435 |
| H | -2.92619 | 3.10452 | -1.09565 |
| H | -4.66978 | -2.4749 | 3.52053 |
| H | -4.44168 | -0.93391 | 2.69605 |
| H | -6.00557 | -1.29134 | 3.43266 |
| H | -4.26415 | -3.30147 | -1.63168 |
| H | -5.71858 | -4.07313 | -0.92844 |
| H | -5.73073 | -2.36257 | -1.36385 |
| H | -1.20685 | 6.85497 | -2.51037 |
| H | -2.79918 | 6.79335 | -3.35347 |
| H | -2.67423 | 7.40149 | -1.61715 |
| H | -8.66099 | 4.90033 | -0.4956 |
| H | -8.64635 | 4.23633 | -2.22203 |
| H | -9.48013 | 3.35065 | -0.89523 |

*o***-DMPB**(c) optimized at the TD B3LYP/6-31G(d) level.

| Atom/ Å | X/ Å | Y/ Å | Z/ Å |
| --- | --- | --- | --- |
| C | -0.40281 | 5.14118 | 0.686 |
| C | -1.73565 | 5.56634 | 0.6172 |
| C | -2.74922 | 4.62994 | 0.41565 |
| C | -2.388 | 3.29831 | 0.265 |
| C | -1.06131 | 2.83381 | 0.31364 |
| C | -0.05998 | 3.79862 | 0.54706 |
| S | -3.55034 | 1.97567 | -0.03752 |
| C | -2.27093 | 0.6575 | 0.42999 |
| C | -0.93689 | 1.38033 | 0.1888 |
| C | 0.19205 | 0.61551 | 0.03608 |
| C | 0.08825 | -0.85413 | 0.06186 |
| S | 1.70298 | -1.57262 | 0.18013 |
| C | 2.48634 | 0.02754 | -0.01025 |
| C | 1.57023 | 1.02942 | -0.09 |
| C | -1.09607 | -1.50954 | -0.06512 |
| C | -2.31712 | -0.64356 | -0.41889 |
| S | -3.78103 | -1.80163 | -0.07288 |
| C | -2.74683 | -3.25659 | -0.16235 |
| C | -1.37939 | -2.94385 | -0.08043 |
| C | -3.23662 | -4.55302 | -0.23873 |
| C | -2.31901 | -5.60354 | -0.21778 |
| C | -0.94908 | -5.32971 | -0.12166 |
| C | -0.47339 | -4.02201 | -0.05515 |
| C | 3.95096 | 0.08355 | -0.05283 |
| O | -3.88151 | 1.952 | -1.47184 |
| O | -4.73335 | -1.77177 | -1.19146 |
| O | -4.29246 | -1.6734 | 1.30151 |
| C | 4.66411 | 1.18844 | -0.60132 |
| C | 6.05715 | 1.16474 | -0.62869 |
| C | 6.77949 | 0.07947 | -0.12133 |
| C | 6.09621 | -1.00719 | 0.42417 |
| C | 4.69861 | -0.9899 | 0.44881 |
| C | -2.41429 | 0.45812 | 1.96059 |
| C | -2.30809 | -0.44363 | -1.95507 |
| O | 6.68283 | -2.12068 | 0.95564 |
| O | 3.92432 | 2.22949 | -1.08903 |
| C | 8.0995 | -2.18836 | 0.96126 |
| C | 4.59524 | 3.29184 | -1.75091 |
| O | -4.64029 | 2.01385 | 0.94699 |
| H | 0.38227 | 5.86933 | 0.87057 |
| H | -1.98048 | 6.61763 | 0.73503 |
| H | -3.79423 | 4.92259 | 0.38231 |
| H | 0.97487 | 3.50624 | 0.66609 |
| H | 1.87385 | 2.05009 | -0.24481 |
| H | -4.30463 | -4.73418 | -0.31133 |
| H | -2.66709 | -6.63058 | -0.27377 |
| H | -0.2382 | -6.15103 | -0.10517 |
| H | 0.59497 | -3.85233 | -0.00042 |
| H | 6.60716 | 1.99866 | -1.04777 |
| H | 7.86218 | 0.10474 | -0.16041 |
| H | 4.20019 | -1.84392 | 0.8954 |
| H | -3.41466 | 0.12657 | 2.22865 |
| H | -1.68906 | -0.28894 | 2.29528 |
| H | -2.20004 | 1.39914 | 2.4733 |
| H | -2.14877 | -1.4074 | -2.44559 |
| H | -3.24297 | -0.01614 | -2.30929 |
| H | -1.4862 | 0.22312 | -2.22852 |
| H | 8.35186 | -3.14366 | 1.42467 |
| H | 8.50969 | -2.1607 | -0.05729 |
| H | 8.54071 | -1.37272 | 1.5499 |
| H | 5.26189 | 3.83542 | -1.06887 |
| H | 5.17264 | 2.92674 | -2.60955 |
| H | 3.81124 | 3.9649 | -2.1021 |

*m***-DMPB**(o) optimized at the B3LYP/6-31G(d) level.

| Atom/ Å | X/ Å | Y/ Å | Z/ Å |
| --- | --- | --- | --- |
| C | -9.99748 | -3.78687 | -1.02686 |
| C | -10.76023 | -3.8658 | -2.12753 |
| C | -10.71263 | -2.88124 | -3.03681 |
| C | -9.9293 | -1.81542 | -2.81274 |
| C | -9.19948 | -1.69527 | -1.6934 |
| C | -9.21189 | -2.71756 | -0.81856 |
| S | -9.7339 | -0.52155 | -4.04346 |
| C | -8.67539 | 0.17703 | -2.76499 |
| C | -8.47694 | -0.55805 | -1.64948 |
| C | -5.55928 | 0.0262 | -2.75311 |
| C | -5.67306 | 0.76737 | -1.62269 |
| C | -6.53698 | 0.51257 | -0.60823 |
| C | -7.71822 | -0.14354 | -0.61293 |
| S | -4.29352 | 0.48309 | -3.95036 |
| C | -3.99729 | 1.78713 | -2.75271 |
| C | -4.81804 | 1.81147 | -1.69261 |
| C | -3.06908 | 2.73085 | -2.97125 |
| C | -2.97033 | 3.7653 | -2.12442 |
| C | -3.84668 | 3.8672 | -1.11495 |
| C | -4.77309 | 2.91436 | -0.92261 |
| S | -6.27991 | 0.98528 | 0.7585 |
| C | -7.3284 | 0.32262 | 1.53786 |
| C | -8.13392 | -0.28588 | 0.65573 |
| C | -7.46223 | 0.38212 | 2.88386 |
| C | -8.45873 | -0.25618 | 3.52989 |
| C | -8.62804 | -0.2543 | 4.86654 |
| C | -7.7272 | 0.4465 | 5.57897 |
| C | -6.73896 | 1.15724 | 5.0087 |
| C | -6.60411 | 1.06115 | 3.67343 |
| O | -10.95364 | 0.26192 | -4.18116 |
| O | -8.90152 | -0.95256 | -5.15811 |
| O | -3.20525 | -0.48479 | -3.92744 |
| O | -4.89928 | 1.01314 | -5.16385 |
| C | -8.2541 | 1.61279 | -2.9813 |
| C | -6.25251 | -1.28187 | -3.07234 |
| O | -5.8089 | 1.81567 | 5.77411 |
| C | -6.08014 | 3.19008 | 5.93017 |
| O | -9.66674 | -0.98182 | 5.39953 |
| C | -9.78125 | -1.0806 | 6.80012 |
| H | -10.00122 | -4.61727 | -0.30044 |
| H | -11.39229 | -4.7523 | -2.30252 |
| H | -11.30073 | -2.96381 | -3.9656 |
| H | -8.57021 | -2.73177 | 0.07559 |
| H | -2.40801 | 2.68003 | -3.85198 |
| H | -2.22315 | 4.55759 | -2.29605 |
| H | -3.82524 | 4.75553 | -0.46103 |
| H | -5.51509 | 3.10324 | -0.13499 |
| H | -9.07527 | -0.78012 | 0.92566 |
| H | -9.20178 | -0.85358 | 2.9793 |
| H | -7.77739 | 0.46996 | 6.67872 |
| H | -5.73288 | 1.59252 | 3.25533 |
| H | -9.11178 | 2.21344 | -3.35939 |
| H | -7.93291 | 2.13153 | -2.0536 |
| H | -7.44867 | 1.68257 | -3.74549 |
| H | -5.77678 | -1.81183 | -3.92746 |
| H | -6.18959 | -2.00294 | -2.22702 |
| H | -7.30944 | -1.11285 | -3.37828 |
| H | -5.28214 | 3.63165 | 6.56793 |
| H | -6.07554 | 3.69572 | 4.93863 |
| H | -7.06527 | 3.32791 | 6.42985 |
| H | -10.65877 | -1.72777 | 7.02423 |
| H | -8.87489 | -1.56318 | 7.22931 |
| H | -9.96899 | -0.07819 | 7.24534 |

*m***-DMPB**(o) optimized at the TD B3LYP/6-31G(d) level.

| Atom/ Å | X/ Å | Y/ Å | Z/ Å |
| --- | --- | --- | --- |
| C | 0.21485 | 4.70723 | -1.59602 |
| C | 1.22449 | 5.55475 | -1.1369 |
| C | 2.14503 | 5.09863 | -0.18469 |
| C | 2.00099 | 3.80444 | 0.28309 |
| C | 0.99122 | 2.93613 | -0.15008 |
| C | 0.09271 | 3.39808 | -1.11426 |
| S | 3.06344 | 2.94375 | 1.43972 |
| C | 2.07846 | 1.43069 | 1.3469 |
| C | 1.05201 | 1.58393 | 0.48261 |
| C | 2.48951 | -0.98381 | -1.30707 |
| C | 1.51878 | -1.44359 | -0.48419 |
| C | 0.25397 | -0.74309 | -0.21823 |
| C | 0.02222 | 0.56837 | 0.17313 |
| S | 3.87906 | -2.13727 | -1.36083 |
| C | 3.07091 | -3.29013 | -0.25579 |
| C | 1.8348 | -2.76504 | 0.14226 |
| C | 3.57634 | -4.49021 | 0.21203 |
| C | 2.80038 | -5.21364 | 1.12633 |
| C | 1.57044 | -4.7091 | 1.55073 |
| C | 1.08252 | -3.48729 | 1.07103 |
| S | -1.26603 | -1.58153 | -0.47692 |
| C | -2.20835 | -0.17953 | -0.02779 |
| C | -1.37426 | 0.86658 | 0.27937 |
| C | -3.67788 | -0.21232 | -0.01429 |
| C | -4.39271 | 0.9643 | -0.24342 |
| C | -5.79591 | 0.94981 | -0.21823 |
| C | -6.48496 | -0.23776 | 0.0196 |
| C | -5.76099 | -1.42108 | 0.24048 |
| C | -4.36662 | -1.41883 | 0.23024 |
| O | 2.96689 | 3.51315 | 2.7936 |
| O | 4.39199 | 2.72506 | 0.84467 |
| O | 4.00819 | -2.68483 | -2.72075 |
| O | 5.0592 | -1.54937 | -0.70673 |
| C | 2.5051 | 0.2997 | 2.21789 |
| C | 2.59624 | 0.23204 | -2.16021 |
| O | -6.52782 | -2.52496 | 0.46716 |
| C | -5.87639 | -3.76758 | 0.68587 |
| O | -6.39468 | 2.15076 | -0.45225 |
| C | -7.81396 | 2.20716 | -0.45442 |
| H | -0.48768 | 5.06441 | -2.34367 |
| H | 1.30358 | 6.56675 | -1.52251 |
| H | 2.94715 | 5.73636 | 0.17397 |
| H | -0.69157 | 2.75086 | -1.49294 |
| H | 4.54408 | -4.85488 | -0.11842 |
| H | 3.16244 | -6.16267 | 1.5101 |
| H | 0.97977 | -5.2687 | 2.27048 |
| H | 0.12988 | -3.10964 | 1.42543 |
| H | -1.74135 | 1.8229 | 0.63347 |
| H | -3.88982 | 1.89782 | -0.46825 |
| H | -7.566 | -0.29153 | 0.04004 |
| H | -3.80743 | -2.32297 | 0.43587 |
| H | 2.70991 | 0.66649 | 3.23075 |
| H | 1.73186 | -0.47047 | 2.26891 |
| H | 3.42891 | -0.15496 | 1.83771 |
| H | 2.95572 | -0.04587 | -3.15814 |
| H | 1.62917 | 0.73102 | -2.2571 |
| H | 3.31691 | 0.94236 | -1.73495 |
| H | -6.67273 | -4.501 | 0.82334 |
| H | -5.2606 | -4.05621 | -0.17573 |
| H | -5.24922 | -3.74037 | 1.58652 |
| H | -8.07021 | 3.24786 | -0.6598 |
| H | -8.24053 | 1.566 | -1.23662 |
| H | -8.22943 | 1.91553 | 0.51877 |

*m***-DMPB**(c) optimized at the B3LYP/6-31G(d) level.

| Atom/ Å | X/ Å | Y/ Å | Z/ Å |
| --- | --- | --- | --- |
| C | -11.59054 | -2.50399 | -1.15705 |
| C | -11.54754 | -3.83811 | -1.03862 |
| C | -10.41182 | -4.42198 | -0.63209 |
| C | -9.34352 | -3.67054 | -0.31813 |
| C | -9.38586 | -2.32855 | -0.38631 |
| C | -10.51565 | -1.76144 | -0.84642 |
| S | -7.80999 | -4.49592 | 0.13098 |
| C | -7.15442 | -2.77144 | 0.37315 |
| C | -8.21953 | -1.76128 | -0.02981 |
| C | -5.91122 | -2.37285 | -0.47637 |
| C | -5.53422 | -0.97439 | -0.0019 |
| C | -6.55442 | -0.09872 | 0.13354 |
| C | -7.84653 | -0.46671 | 0.05278 |
| S | -4.4284 | -3.49071 | -0.34628 |
| C | -3.53074 | -1.96802 | -0.01406 |
| C | -4.20592 | -0.81802 | 0.15939 |
| C | -2.19464 | -2.02302 | 0.11354 |
| C | -1.50394 | -0.91927 | 0.43038 |
| C | -2.16261 | 0.23205 | 0.62046 |
| C | -3.49829 | 0.27861 | 0.4894 |
| S | -6.4686 | 1.34744 | 0.31649 |
| C | -7.873 | 1.78097 | 0.22774 |
| C | -8.58742 | 0.64854 | 0.09373 |
| C | -8.28461 | 3.07798 | 0.31354 |
| C | -9.58567 | 3.46406 | 0.19395 |
| C | -9.85947 | 4.78082 | 0.29485 |
| C | -8.92486 | 5.71056 | 0.51596 |
| C | -7.63323 | 5.37658 | 0.6413 |
| C | -7.37256 | 4.05974 | 0.5309 |
| O | -8.00624 | -5.18107 | 1.39986 |
| O | -7.14527 | -5.0724 | -1.022 |
| O | -4.03007 | -4.00308 | -1.64888 |
| O | -4.57892 | -4.30094 | 0.84733 |
| C | -6.97152 | -2.64221 | 1.90881 |
| C | -6.17926 | -2.25089 | -2.00059 |
| O | -6.71501 | 6.37584 | 0.86685 |
| C | -5.35459 | 6.04368 | 1.0219 |
| O | -10.58294 | 2.55246 | -0.05538 |
| C | -11.89816 | 2.97712 | -0.32652 |
| H | -12.50912 | -2.01998 | -1.53058 |
| H | -12.4251 | -4.45005 | -1.30557 |
| H | -10.36526 | -5.52245 | -0.57463 |
| H | -10.61801 | -0.68347 | -1.01694 |
| H | -1.65096 | -2.97055 | -0.03828 |
| H | -0.4073 | -0.95883 | 0.53844 |
| H | -1.60024 | 1.14261 | 0.88922 |
| H | -3.9578 | 1.25852 | 0.67172 |
| H | -9.67286 | 0.59735 | 0.08131 |
| H | -10.88769 | 5.16342 | 0.19927 |
| H | -9.21712 | 6.77204 | 0.59254 |
| H | -6.30844 | 3.81023 | 0.63861 |
| H | -6.28944 | -3.39901 | 2.34863 |
| H | -6.55594 | -1.66135 | 2.22412 |
| H | -7.94328 | -2.72969 | 2.44748 |
| H | -5.302 | -1.83436 | -2.5471 |
| H | -6.44303 | -3.21247 | -2.48811 |
| H | -7.01592 | -1.56093 | -2.24386 |
| H | -4.79354 | 6.98843 | 1.20035 |
| H | -5.21402 | 5.3867 | 1.90917 |
| H | -4.95925 | 5.57954 | 0.09087 |
| H | -12.50383 | 2.0696 | -0.54855 |
| H | -12.34204 | 3.46993 | 0.56688 |
| H | -11.91879 | 3.63138 | -1.2265 |

*m***-DMPB**(c) optimized at the TD B3LYP/6-31G(d) level.

| C | 0.22654 | -5.17004 | 0.71673 |
| --- | --- | --- | --- |
| C | 1.53226 | -5.66974 | 0.63346 |
| C | 2.59424 | -4.79315 | 0.41318 |
| C | 2.30662 | -3.44429 | 0.25731 |
| C | 1.00882 | -2.90557 | 0.31842 |
| C | -0.04132 | -3.81106 | 0.57295 |
| S | 3.53874 | -2.19221 | -0.06694 |
| C | 2.33978 | -0.79927 | 0.39854 |
| C | 0.96423 | -1.44817 | 0.181 |
| C | 2.45112 | 0.48676 | -0.46676 |
| C | 1.28596 | 1.42613 | -0.11258 |
| C | 0.06819 | 0.84111 | 0.03745 |
| C | -0.12066 | -0.62192 | 0.03121 |
| S | 3.98261 | 1.56259 | -0.14903 |
| C | 3.03307 | 3.07348 | -0.2521 |
| C | 1.65076 | 2.84172 | -0.1533 |
| C | 3.59615 | 4.33795 | -0.3542 |
| C | 2.74098 | 5.44002 | -0.34287 |
| C | 1.35847 | 5.24754 | -0.23138 |
| C | 0.80883 | 3.97074 | -0.13924 |
| S | -1.50412 | 1.65764 | 0.15826 |
| C | -2.37079 | 0.1094 | -7.20E-04 |
| C | -1.52539 | -0.95009 | -0.06928 |
| C | -3.83821 | 0.09757 | -0.05157 |
| C | -4.49631 | -0.90624 | -0.765 |
| C | -5.89864 | -0.93472 | -0.80215 |
| C | -6.64153 | 0.04035 | -0.13845 |
| C | -5.97437 | 1.05249 | 0.57111 |
| C | -4.58116 | 1.09107 | 0.61993 |
| O | 4.63222 | -2.28175 | 0.91009 |
| O | 3.85923 | -2.1992 | -1.50362 |
| O | 4.92185 | 1.46192 | -1.27416 |
| O | 4.49599 | 1.42509 | 1.22361 |
| C | 2.51093 | -0.59112 | 1.92514 |
| C | 2.41363 | 0.2704 | -2.00044 |
| O | -6.79188 | 1.95201 | 1.18675 |
| C | -6.19823 | 3.01371 | 1.92029 |
| O | -6.44172 | -1.95277 | -1.52559 |
| C | -7.85661 | -2.02857 | -1.62737 |
| H | -0.59614 | -5.85117 | 0.9156 |
| H | 1.719 | -6.73244 | 0.75473 |
| H | 3.62056 | -5.14454 | 0.3689 |
| H | -1.05652 | -3.46169 | 0.7063 |
| H | 4.67206 | 4.45576 | -0.43923 |
| H | 3.14723 | 6.44412 | -0.41893 |
| H | 0.69646 | 6.10885 | -0.22382 |
| H | -0.26717 | 3.86443 | -0.07522 |
| H | -1.89705 | -1.95881 | -0.173 |
| H | -3.9483 | -1.65518 | -1.32508 |
| H | -7.72397 | 0.05628 | -0.14916 |
| H | -4.06474 | 1.85152 | 1.19209 |
| H | 3.53157 | -0.31472 | 2.1789 |
| H | 1.83351 | 0.2 | 2.25888 |
| H | 2.24906 | -1.51265 | 2.45094 |
| H | 2.30479 | 1.23603 | -2.50097 |
| H | 3.31842 | -0.21437 | -2.35887 |
| H | 1.55188 | -0.35097 | -2.25761 |
| H | -7.02773 | 3.60646 | 2.30942 |
| H | -5.5968 | 2.6372 | 2.75777 |
| H | -5.57023 | 3.6443 | 1.27784 |
| H | -8.06382 | -2.90195 | -2.24791 |
| H | -8.32409 | -2.16233 | -0.64329 |
| H | -8.27333 | -1.13377 | -2.10736 |
| C | 0.22654 | -5.17004 | 0.71673 |

*p***-DMPB**(o) optimized at the B3LYP/6-31G(d) level.

| Atom/ Å | X/ Å | Y/ Å | Z/ Å |
| --- | --- | --- | --- |
| C | -1.03441 | -4.69774 | 1.36985 |
| C | -2.27695 | -4.97844 | 0.95473 |
| C | -2.94588 | -4.05643 | 0.25019 |
| C | -2.3469 | -2.89542 | -0.05686 |
| C | -1.08408 | -2.60325 | 0.29775 |
| C | -0.45796 | -3.52501 | 1.05744 |
| S | -3.2834 | -1.6003 | -0.87525 |
| C | -1.68139 | -0.78239 | -0.79976 |
| C | -0.66912 | -1.38618 | -0.13247 |
| C | 0.55544 | -0.82361 | 0.02451 |
| C | 0.9193 | 0.47683 | 0.06354 |
| C | 2.84515 | -0.65712 | 0.09452 |
| C | 0.16599 | 1.59021 | 0.18222 |
| C | -0.89623 | 1.6817 | 1.01142 |
| S | -1.74165 | 3.26858 | 1.10737 |
| C | -0.42893 | 3.74625 | -0.02321 |
| C | 0.41666 | 2.77731 | -0.40493 |
| C | -0.3456 | 4.99259 | -0.51272 |
| C | 0.59324 | 5.28006 | -1.4261 |
| C | 1.42544 | 4.31529 | -1.84557 |
| C | 1.33286 | 3.07226 | -1.34535 |
| C | -1.55259 | 0.38295 | -1.75765 |
| C | -1.33019 | 0.64898 | 2.02576 |
| O | -4.20942 | -0.96863 | 0.05434 |
| O | -3.61901 | -1.99302 | -2.23672 |
| O | -3.01556 | 3.20764 | 0.40438 |
| O | -1.54401 | 3.89112 | 2.4089 |
| C | 4.1691 | -0.93745 | 0.10764 |
| C | 4.65711 | -2.19355 | 0.19065 |
| C | 5.10351 | 0.03023 | 0.03967 |
| C | 5.96797 | -2.50536 | 0.23059 |
| C | 6.41258 | -0.25719 | 0.01667 |
| C | 6.87148 | -1.52006 | 0.03932 |
| C | 2.2603 | 0.54815 | 0.05737 |
| S | 1.76543 | -1.6429 | 0.1746 |
| O | 8.22066 | -1.77147 | 0.05335 |
| O | 6.36543 | -3.81946 | 0.28043 |
| C | 8.7582 | -2.05095 | -1.21928 |
| C | 6.60387 | -4.28852 | 1.58795 |
| H | -0.49246 | -5.43337 | 1.98887 |
| H | -2.75575 | -5.93438 | 1.22387 |
| H | -3.98796 | -4.25399 | -0.05118 |
| H | 0.5434 | -3.38054 | 1.48371 |
| H | -1.04186 | 5.77821 | -0.17607 |
| H | 0.66764 | 6.29799 | -1.84355 |
| H | 2.17675 | 4.54264 | -2.62087 |
| H | 2.00594 | 2.31022 | -1.7672 |
| H | -2.06875 | 0.15752 | -2.7177 |
| H | -2.03842 | 1.29902 | -1.35865 |
| H | -0.50806 | 0.59072 | -2.07172 |
| H | -1.53862 | 1.12986 | 3.00785 |
| H | -2.26922 | 0.14766 | 1.70466 |
| H | -0.55547 | -0.1163 | 2.24336 |
| H | 3.97784 | -3.06092 | 0.24775 |
| H | 4.8458 | 1.09905 | -0.01407 |
| H | 7.14085 | 0.5705 | -0.04076 |
| H | 2.81308 | 1.49484 | 0.09837 |
| H | 9.8487 | -2.23915 | -1.10166 |
| H | 8.27715 | -2.95735 | -1.64947 |
| H | 8.60867 | -1.17839 | -1.89404 |
| H | 7.41605 | -3.68845 | 2.05553 |
| H | 5.67084 | -4.22232 | 2.19127 |
| H | 6.91018 | -5.36719 | 1.54885 |

*p***-DMPB**(o) optimized at the TD B3LYP/6-31G(d) level.

| Atom/ Å | X/ Å | Y/ Å | Z/ Å |
| --- | --- | --- | --- |
| C | 1.00378 | 4.76117 | 1.56706 |
| C | 2.15945 | 5.43047 | 1.16235 |
| C | 3.03916 | 4.82432 | 0.25679 |
| C | 2.70807 | 3.56931 | -0.2226 |
| C | 1.54946 | 2.87881 | 0.15563 |
| C | 0.6933 | 3.48693 | 1.07616 |
| S | 3.6801 | 2.54447 | -1.32133 |
| C | 2.45977 | 1.21254 | -1.29274 |
| C | 1.42464 | 1.53058 | -0.48132 |
| C | 0.26375 | 0.6627 | -0.23652 |
| C | 0.2074 | -0.67143 | 0.14254 |
| C | -2.10239 | -0.22887 | -0.0745 |
| C | 1.36094 | -1.54189 | 0.45781 |
| C | 2.34623 | -1.26046 | 1.33762 |
| S | 3.52224 | -2.63017 | 1.43553 |
| C | 2.6004 | -3.61419 | 0.25679 |
| C | 1.49009 | -2.88424 | -0.18538 |
| C | 2.92285 | -4.87298 | -0.21857 |
| C | 2.0856 | -5.4387 | -1.18868 |
| C | 0.9787 | -4.72897 | -1.65722 |
| C | 0.67559 | -3.45269 | -1.16716 |
| C | 2.74232 | 0.02858 | -2.1506 |
| C | 2.60671 | -0.08995 | 2.22178 |
| O | 4.92043 | 2.11772 | -0.6536 |
| O | 3.75198 | 3.11285 | -2.67691 |
| O | 4.81871 | -2.23336 | 0.86254 |
| O | 3.48161 | -3.21885 | 2.78395 |
| C | -3.56072 | -0.39368 | -0.07404 |
| C | -4.42106 | 0.6955 | 0.14674 |
| C | -4.13225 | -1.66145 | -0.2813 |
| C | -5.8045 | 0.53719 | 0.1514 |
| C | -5.51405 | -1.82405 | -0.28308 |
| C | -6.36444 | -0.73448 | -0.08321 |
| C | -1.13682 | -1.15619 | 0.23239 |
| S | -1.35312 | 1.29074 | -0.50526 |
| O | -7.72163 | -0.94424 | -0.07632 |
| O | -6.59211 | 1.64661 | 0.35514 |
| C | -8.44116 | -0.32979 | -1.15519 |
| C | -7.31427 | 1.65565 | 1.5944 |
| H | 0.33197 | 5.23153 | 2.27941 |
| H | 2.38346 | 6.418 | 1.5543 |
| H | 3.95261 | 5.31906 | -0.05867 |
| H | -0.20451 | 2.98234 | 1.41527 |
| H | 3.79783 | -5.40125 | 0.14758 |
| H | 2.30504 | -6.42712 | -1.581 |
| H | 0.34191 | -5.16923 | -2.41935 |
| H | -0.18208 | -2.91165 | -1.55339 |
| H | 3.07356 | 0.36002 | -3.14194 |
| H | 3.54752 | -0.57999 | -1.71921 |
| H | 1.85369 | -0.59682 | -2.26353 |
| H | 2.84646 | -0.43448 | 3.23478 |
| H | 3.46584 | 0.48739 | 1.85679 |
| H | 1.73679 | 0.56946 | 2.26758 |
| H | -4.02894 | 1.68872 | 0.34423 |
| H | -3.49327 | -2.51742 | -0.47453 |
| H | -5.96194 | -2.79756 | -0.45761 |
| H | -1.37205 | -2.15621 | 0.57819 |
| H | -9.49135 | -0.58513 | -0.99944 |
| H | -8.31599 | 0.75709 | -1.15053 |
| H | -8.10645 | -0.7344 | -2.11866 |
| H | -7.99454 | 0.80131 | 1.66608 |
| H | -6.62001 | 1.64443 | 2.44418 |
| H | -7.88444 | 2.58695 | 1.60378 |

*p***-DMPB**(c) optimized at the B3LYP/6-31G(d) level.

| Atom/ Å | X/ Å | Y/ Å | Z/ Å |
| --- | --- | --- | --- |
| C | -10.01506 | -1.22334 | 0.38483 |
| C | -10.36741 | -2.34021 | 1.03611 |
| C | -9.41207 | -3.11962 | 1.56137 |
| C | -8.12087 | -2.76342 | 1.459 |
| C | -7.75463 | -1.62307 | 0.84849 |
| C | -8.72129 | -0.87831 | 0.28292 |
| S | -6.86354 | -3.88142 | 2.09469 |
| C | -5.66622 | -2.56251 | 1.55744 |
| C | -6.42149 | -1.4455 | 0.85103 |
| C | -5.65485 | -0.44742 | 0.36303 |
| C | -4.3237 | -0.58452 | 0.19415 |
| S | -3.73776 | 0.68007 | -0.25134 |
| C | -4.92869 | 1.52635 | -0.38916 |
| C | -5.98991 | 0.79532 | -0.00979 |
| C | -3.68405 | -1.76353 | 0.36305 |
| C | -4.57371 | -2.98946 | 0.53309 |
| S | -3.52716 | -4.45573 | 1.00561 |
| C | -2.14671 | -3.36823 | 0.62374 |
| C | -2.37176 | -2.06962 | 0.35587 |
| C | -0.90162 | -3.87019 | 0.6721 |
| C | 0.15359 | -3.07157 | 0.46185 |
| C | -0.05135 | -1.77204 | 0.20801 |
| C | -1.29964 | -1.2792 | 0.1598 |
| C | -4.92056 | 2.81648 | -0.80102 |
| O | -6.98965 | -3.94049 | 3.5434 |
| O | -6.68301 | -5.03552 | 1.23493 |
| O | -3.60054 | -5.50675 | 0.00164 |
| O | -3.67546 | -4.66765 | 2.4328 |
| C | -6.05223 | 3.53882 | -0.9121 |
| C | -6.03302 | 4.82497 | -1.28936 |
| C | -4.89009 | 5.47706 | -1.56161 |
| C | -3.75226 | 4.75023 | -1.58311 |
| C | -3.79324 | 3.47665 | -1.14318 |
| C | -5.12752 | -1.9685 | 2.88612 |
| C | -5.11024 | -3.33036 | -0.88321 |
| O | -4.91554 | 6.78286 | -1.98338 |
| O | -2.54756 | 5.34461 | -1.87095 |
| C | -2.18818 | 5.26448 | -3.23144 |
| C | -4.7011 | 7.71649 | -0.94949 |
| H | -10.79388 | -0.59809 | -0.08424 |
| H | -11.42836 | -2.63142 | 1.10975 |
| H | -9.70124 | -4.0587 | 2.06234 |
| H | -8.5131 | 0.02329 | -0.30417 |
| H | -7.00329 | 1.19635 | 0.04604 |
| H | -0.73084 | -4.93877 | 0.88528 |
| H | 1.17804 | -3.47738 | 0.50244 |
| H | 0.8137 | -1.10733 | 0.04223 |
| H | -1.37402 | -0.20336 | -0.04358 |
| H | -7.0467 | 3.11979 | -0.69545 |
| H | -6.98752 | 5.37441 | -1.36574 |
| H | -2.80914 | 2.98094 | -1.09105 |
| H | -4.63688 | -2.71168 | 3.54823 |
| H | 4.3703 | -1.16905 | 2.73861 |
| H | -5.93897 | -1.48978 | 3.48154 |
| H | -4.28518 | -3.49775 | -1.61329 |
| H | -5.76148 | -4.22868 | -0.91168 |
| H | -5.71889 | -2.51431 | -1.32911 |
| H | -2.08974 | 4.19847 | -3.53624 |
| H | -2.95052 | 5.77643 | -3.85999 |
| H | -1.20722 | 5.77354 | -3.36165 |
| H | -4.74179 | 8.73756 | -1.39014 |
| H | -5.49853 | 7.61889 | -0.17905 |
| H | -3.70016 | 7.55822 | -0.49004 |

*p***-DMPB**(c) optimized at the TD B3LYP/6-31G(d) level.

| Atom/ Å | X/ Å | Y/ Å | Z/ Å |
| --- | --- | --- | --- |
| C | 0.77558 | -5.257 | 0.37551 |
| C | 2.12176 | -5.63579 | 0.29881 |
| C | 3.10694 | -4.65888 | 0.15723 |
| C | 2.70507 | -3.3333 | 0.07 |
| C | 1.36378 | -2.91385 | 0.12596 |
| C | 0.39225 | -3.92023 | 0.30144 |
| S | 3.82787 | -1.96075 | -0.14743 |
| C | 2.50051 | -0.70949 | 0.36995 |
| C | 1.19399 | -1.46041 | 0.07141 |
| C | 0.0442 | -0.72463 | -0.06585 |
| C | 0.10079 | 0.74563 | 0.03691 |
| S | -1.5411 | 1.41128 | 0.15295 |
| C | -2.26155 | -0.19281 | -0.13643 |
| C | -1.32083 | -1.16752 | -0.23756 |
| C | 1.26507 | 1.4435 | -0.03253 |
| C | 2.51833 | 0.63521 | -0.409 |
| S | 3.93964 | 1.81802 | 0.02211 |
| C | 2.86108 | 3.24303 | -0.00826 |
| C | 1.50304 | 2.88493 | 0.03304 |
| C | 3.31109 | 4.55602 | -0.00709 |
| C | 2.36075 | 5.5754 | 0.05384 |
| C | 0.99876 | 5.25533 | 0.11161 |
| C | 0.56347 | 3.93201 | 0.10007 |
| C | -3.71634 | -0.32806 | -0.24748 |
| O | 4.90536 | -2.01514 | 0.84963 |
| O | 4.17549 | -1.85106 | -1.57361 |
| O | 4.90859 | 1.87664 | -1.08061 |
| O | 4.43334 | 1.63336 | 1.39634 |
| C | -4.28321 | -1.39102 | -0.97473 |
| C | -5.66356 | -1.53788 | -1.0528 |
| C | -6.51853 | -0.62703 | -0.42643 |
| C | -5.96491 | 0.46718 | 0.26724 |
| C | -4.58174 | 0.59983 | 0.35788 |
| C | 2.61641 | -0.58655 | 1.91085 |
| C | 2.53912 | 0.51725 | -1.95368 |
| O | -7.87441 | -0.79588 | -0.55521 |
| O | -6.75828 | 1.3928 | 0.90469 |
| C | -7.50013 | 2.25382 | 0.02928 |
| C | -8.57396 | -1.09791 | 0.66123 |
| H | 0.01233 | -6.01753 | 0.51484 |
| H | 2.39883 | -6.68351 | 0.36521 |
| H | 4.16099 | -4.9167 | 0.12156 |
| H | -0.65207 | -3.66761 | 0.42948 |
| H | -1.59135 | -2.19741 | -0.41856 |
| H | 4.37387 | 4.77336 | -0.05128 |
| H | 2.67731 | 6.61405 | 0.05866 |
| H | 0.26242 | 6.05257 | 0.15972 |
| H | -0.49991 | 3.72796 | 0.12779 |
| H | -3.64112 | -2.09609 | -1.49289 |
| H | -6.10809 | -2.35835 | -1.60772 |
| H | -4.19524 | 1.43767 | 0.93039 |
| H | 3.60256 | -0.24043 | 2.21121 |
| H | 1.86471 | 0.12061 | 2.2729 |
| H | 2.4234 | -1.5589 | 2.37075 |
| H | 2.35487 | 1.49996 | -2.39543 |
| H | 3.49325 | 0.14047 | -2.31398 |
| H | 1.74489 | -0.16101 | -2.2761 |
| H | -6.81962 | 2.84643 | -0.59504 |
| H | -8.17966 | 1.68194 | -0.61043 |
| H | -8.0725 | 2.92095 | 0.67713 |
| H | -9.62644 | -1.19153 | 0.38615 |
| H | -8.22043 | -2.04842 | 1.0801 |
| H | -8.4497 | -0.30185 | 1.40116 |
